# Effects of the Informed Health Choices primary school intervention on the ability of children in Uganda to assess the reliability of claims about treatment effects, 1-year follow-up: a cluster-randomised trial

**DOI:** 10.1186/s13063-019-3960-9

**Published:** 2020-01-06

**Authors:** Allen Nsangi, Daniel Semakula, Andrew D. Oxman, Astrid Austvoll-Dahlgren, Matt Oxman, Sarah Rosenbaum, Angela Morelli, Claire Glenton, Simon Lewin, Margaret Kaseje, Iain Chalmers, Atle Fretheim, Yunpeng Ding, Nelson K. Sewankambo

**Affiliations:** 1 College of Health Sciences, Makerere University, Kampala, Uganda; 2 University of Oslo, Oslo, Norway; 3 Centre for Informed Health Choices, Norwegian Institute of Public Health, Postboks 222 Skøyen, 0213 Oslo, Norway; 4 Infodesignlab, Oslo, Norway; 5 Health Systems Research Unit, South African Medical Research Council, Cape Town, South Africa; 6 Tropical Institute of Community Health & Development, Kisumu, Kenya; 7 James Lind Initiative, Oxford, UK

**Keywords:** Evidence-based healthcare, Training, Critical thinking, Health literacy, Informed decision-making, Public involvement, Children

## Abstract

**Introduction:**

We evaluated an intervention designed to teach 10- to 12-year-old primary school children to assess claims about the effects of treatments (any action intended to maintain or improve health). We report outcomes measured 1 year after the intervention.

**Methods:**

In this cluster-randomised trial, we included primary schools in the central region of Uganda that taught year 5 children (aged 10 to 12 years). We randomly allocated a representative sample of eligible schools to either an intervention or control group. Intervention schools received the Informed Health Choices primary school resources (textbooks, exercise books and a teachers’ guide). The primary outcomes, measured at the end of the school term and again after 1 year, were the mean score on a test with two multiple-choice questions for each of the 12 concepts and the proportion of children with passing scores.

**Results:**

We assessed 2960 schools for eligibility; 2029 were eligible, and a random sample of 170 were invited to recruitment meetings. After recruitment meetings, 120 eligible schools consented and were randomly assigned to either the intervention group (*n* = 60 schools; 76 teachers and 6383 children) or the control group (*n* = 60 schools; 67 teachers and 4430 children). After 1 year, the mean score in the multiple-choice test for the intervention schools was 68.7% compared with 53.0% for the control schools (adjusted mean difference 16.7%; 95% CI, 13.9 to 19.5; *P* < 0.00001). In the intervention schools, 3160 (80.1%) of 3943 children who completed the test after 1 year achieved a predetermined passing score (≥ 13 of 24 correct answers) compared with 1464 (51.5%) of 2844 children in the control schools (adjusted difference, 39.5%; 95% CI, 29.9 to 47.5).

**Conclusion:**

Use of the learning resources led to a large improvement in the ability of children to assess claims, which was sustained for at least 1 year.

**Trial registration:**

Pan African Clinical Trial Registry (www.pactr.org), PACTR201606001679337. Registered on 13 June 2016.

Summary Box **What is already known**

- There is an overload of unsubstantiated claims about the benefits and harms of treatments.
- Many people are unable to assess the reliability of these claims.
- This leads to poorly informed decisions, unnecessary suffering and waste.

**What are the new findings**

- Children (aged 10–12 years) who used the Informed Health Choices primary school resources learned to think critically about treatment claims and retained what they learned for at least 1 year.

**How might it impact clinical practice in the foreseeable future?**

- In the short term, children are likely to think more critically about treatment claims and choices.
- In the long term, they may be enabled to make well-informed decisions as patients and future health professionals and as citizens and future policymakers.

Summary Box **What is already known**

- There is an overload of unsubstantiated claims about the benefits and harms of treatments.
- Many people are unable to assess the reliability of these claims.
- This leads to poorly informed decisions, unnecessary suffering and waste.

**What are the new findings**

- Children (aged 10–12 years) who used the Informed Health Choices primary school resources learned to think critically about treatment claims and retained what they learned for at least 1 year.

**How might it impact clinical practice in the foreseeable future?**

- In the short term, children are likely to think more critically about treatment claims and choices.
- In the long term, they may be enabled to make well-informed decisions as patients and future health professionals and as citizens and future policymakers.

## Background

We identified Informed Health Choices (IHC) key concepts that people need to understand and apply when assessing claims about treatments [1, 2]. Together with teachers in Uganda, we determined which of those concepts were relevant for primary school children [3]. We then prototyped, user-tested and piloted learning resources to teach 12 key concepts (Table 1) to children [5], and we developed and validated a test to measure their ability to apply those concepts [6–10].

**Table 1:** Twelve key concepts covered by the Informed Health Choices primary school resources

Claims	
• Treatments may be harmful. • Personal experiences or anecdotes (stories) are an unreliable basis for assessing the effects of most treatments. • Widely used treatments or treatments that have been used for a long time are not necessarily beneficial or safe. • New, brand-named, or more expensive treatments may not be better than available alternatives. • Opinions of experts or authorities do not alone provide a reliable basis for deciding on the benefits and harms of treatments. • Conflicting interests may result in misleading claims about the effects of treatments.	
Comparisons	
• Evaluating the effects of treatments requires appropriate comparisons • Apart from the treatments being compared, the comparison groups need to be similar (i.e., ‘like needs to be compared with like’). • If possible, people should not know which of the treatments being compared they are receiving. • Small studies in which few outcome events occur are usually not informative, and the results may be misleading. • The results of single comparisons of treatments can be misleading.	
Choices	
• Treatments usually have beneficial and harmful effects.	

The concepts are shown here as they are described in the key concepts list [3], which was not designed as a learning resource, not as they were presented to the children in the primary school resources [4]

**Fig. 1:**
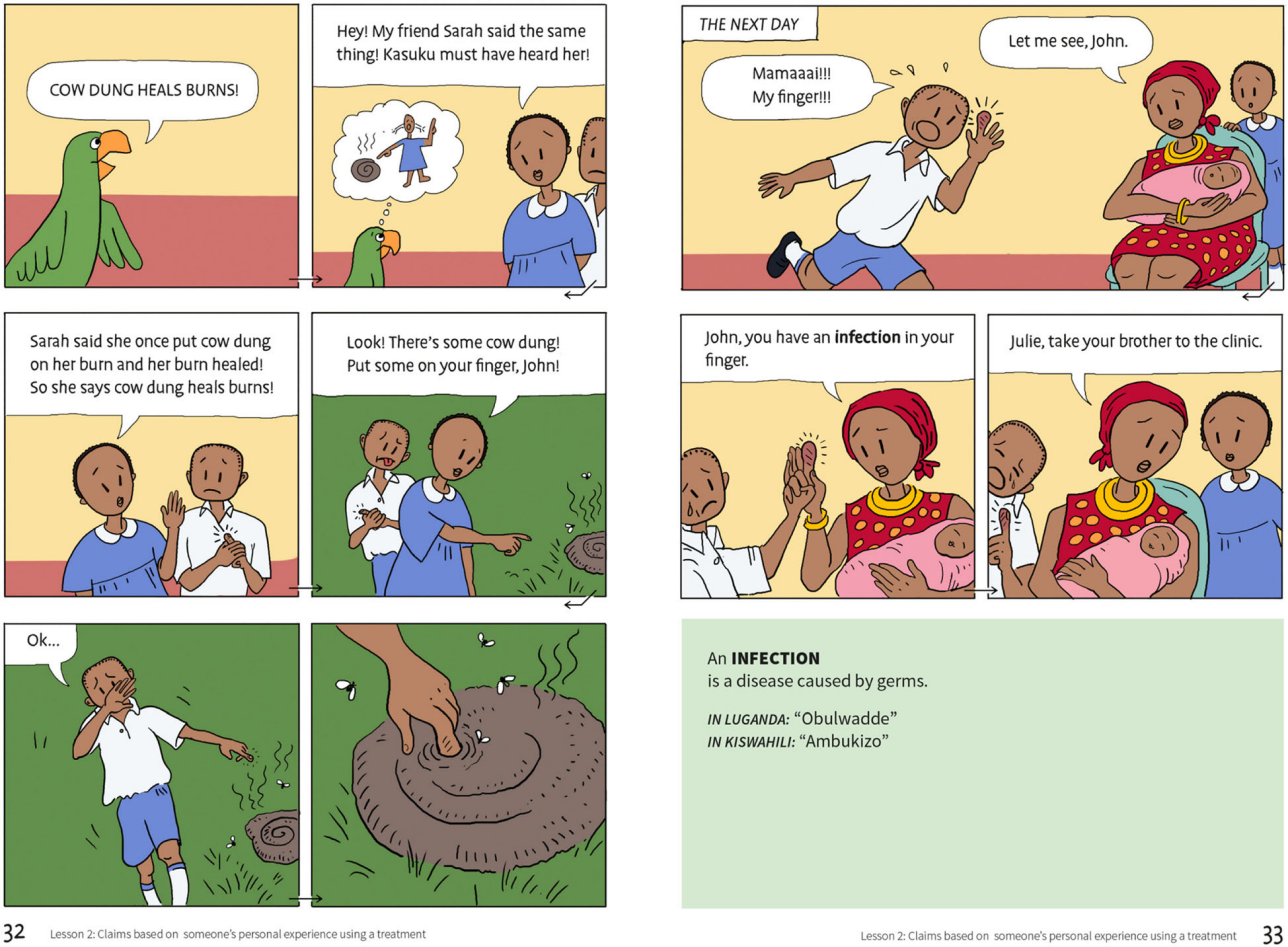
An excerpt from the comic story in the textbook

The resulting learning resources, which were printed in English, included a textbook, a teachers’ guide, an exercise book, a poster, and cards for an activity. The textbook [11] consists of a story in a comic book format (Fig. 1), instructions for classroom activities, exercises, a checklist summarising the concepts in the book, and a glossary of keywords with definitions in English and translations to Luganda and Swahili. In addition to the textbooks, we provided intervention schools with a guide [4] for each teacher, an exercise book for each child, a poster of the checklist for the classroom, and activity cards for the seventh lesson [12]. The contents of the book and the teachers’ guide are shown in Table 2. While most teachers considered the IHC content to be new, many found the design of the IHC lessons to be compatible with their teaching styles, particularly the use of multiple examples in the teachers’ guide [13]. We did not intervene in the control schools.

**Table 2:** Contents of the textbook and the teachers’ guide

Health Choices Book Learning to think carefully about treatments A health science book for primary school children	Teachers’ Guide
Introduction • Lesson 1: Health, treatments and effects of treatments John and Julie learn about CLAIMS about treatments • Lesson 2: Someone’s experience using a treatment • Lesson 3: Other bad bases for claims about treatments (part 1) • Lesson 4: Other bad bases for claims about treatments (part 2) John and Julie learn about COMPARISONS of treatments • Lesson 5: Comparisons of treatments • Lesson 6: Fair comparisons of treatments • Lesson 7: Big-enough fair comparisons of treatments John and Julie learn about CHOICES about treatments • Lesson 8: Advantages and disadvantages of a treatment Review • Lesson 9: Review of what is most important to remember from this book	The teacher’s guide includes an introduction to the project and the resources, and the following for each lesson, in addition to the embedded chapter from the textbook: • The objective of the lesson • A lesson preparation plan • A lesson plan • A list of materials that the teacher and children will need • A synopsis of the story • Keywords in the chapter • Review questions to ask the children after reading the story • Extra examples for illustrating the concepts • Background about examples used in the story • Teacher instructions for the classroom activity • Answers and explanations for the activity • Answers and explanations for the exercises • Background information, examples and keyword definitions for teachers

We conducted a cluster-randomised trial to evaluate the effects of using the learning resources [14, 15]. The intervention included a 2-day introductory workshop for the teachers, as well as providing them with the learning resources. The trial showed that the intervention resulted in a large improvement in the ability of children to assess claims about the effects of treatments, measured at the end of the term during which the intervention was delivered [14]. In this paper, we report on outcomes measured 1 year after the intervention. We report a process evaluation in a separate paper [13].

## Methods

Details regarding the study methods can be found in the trial protocol [15] and report of the initial results [14]. They are briefly summarised here.

### Participants

**Fig. 2:**
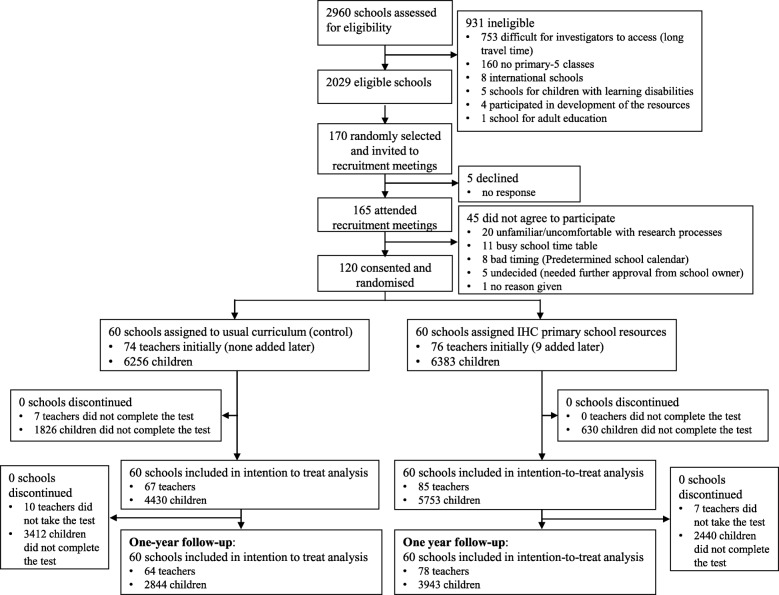
Informed Health Choices trial profile

Between April 11, 2016, and June 8, 2016, we randomly selected 170 of 2029 eligible schools in central Uganda and recruited 120 of those schools (Fig. 2). We randomly sampled schools proportionately from lists of randomly selected districts, stratifying by school ownership (private or public) and location (urban, semi-urban and rural). We excluded international schools, special needs schools for children with visual and audio impairments, schools that had participated in user testing and piloting of the resources, infant and nursery schools and adult education schools. We included all year 5 children in the eligible schools.

### Random allocation and blinding

We randomly allocated schools to the intervention or control group using a computer-generated sequence. We used stratified randomisation to help ensure equal distribution of schools for school ownership (public or private) and geographical location (urban, semi-urban or rural). Research assistants labelled opaque envelopes with the unique codes, inserted cards with the study group allocated to each code in the envelopes, and sealed them. After obtaining consent from 120 schools, 2 research assistants selected each school from a list of the schools; identified the appropriate randomisation list to be used for that school, based on its geographical location and ownership; and assigned the next available code from that list.

We informed the participating head teachers and year 5 teachers about the objectives of the study [15]. After randomisation, they knew whether they were in the intervention or control arm. The consent form stated that the outcome measure consisted of ‘multiple-choice questions that assess an individual’s ability to apply concepts that people must be able to understand and apply to assess treatment claims and to make informed healthcare choices.’ We did not show them the test until the end of the school term. Children in both arms of the trial were informed of the purpose of the test when their teachers asked them to complete it at the end of the term and again after 1 year.

### Interventions

We designed the learning resources to be used over 9 weeks, with one double-period (80 min) per week, during a single school term, and 1 h to complete the test at the end of the term and again after 1 year. There was an expectation on the part of the head teachers and teachers that any content displaced by the lessons would be compensated, so that time was not taken away from other lessons. Each school decided how to do this, with some schools using the library lessons while boarding schools preferred to teach in the evenings and on weekends [13]. The intervention was delivered between June and August 2016.

We invited all participating teachers in the intervention group to attend an introductory workshop. At the workshop, we informed them about the study objectives and procedures, including the general nature of the outcome measure; went through all nine lessons outlined in the primary school resources; and addressed any questions or concerns that arose.

We invited year 5 teachers in the control schools to a 2-h introductory meeting in each district. At these meetings, we informed them about the study procedures, including the general nature of the test that we would be using as the outcome measure. We told them that they would receive the primary school resources at the end of the study. We did not introduce them to the resources.

### Outcomes

The primary outcomes, measured using the same test taken at the end of the term when the intervention was delivered, were as follows:

1. The mean test score (percentage of correct answers) on the same test 1 year later
2. The proportion of children with a passing score

Secondary outcomes were as follows:

1. The proportion of children with a score indicating mastery of the concepts
2. For each concept, the proportion of children who answered both questions correctly
3. The children’s intended behaviours and self-efficacy
4. Self-reported behaviours
5. Mean scores, passing scores and mastery scores for the teachers, who took the same test as the children at the end of the intervention term and again 1 year later

Most teachers completed the test at the same time as the children. We contacted teachers who were not available on the day of the exam to arrange completion of the questionnaire on another day. The children and the teachers were aware that missing answers would be scored as wrong.

The test included 24 multiple-choice questions (2 for each concept) (Additional file 1) [9]. The questions had between two and four response options, with an overall probability of answering 39% of the questions correctly by chance alone. Two additional multiple-choice questions were included because the test used in this trial was also used in a linked randomised trial evaluating a podcast given to the parents of some of the children [16]. These two extra questions were not included in the primary analyses.

The test also included questions that assessed intended behaviours, self-efficacy, attitudes and reading skills (Additional file 1). For questions about intended behaviours and self-efficacy, we dichotomised the responses (e.g., very unlikely or unlikely versus very likely or likely) in the analysis, and we reported the proportions of children for each of the four responses. We used the answers to the reading skills questions as a covariate in exploratory analyses. In the test taken after 1 year, we also collected data on self-reported behaviours (Table 3). We made the comparisons shown in Additional file 2: Table S1 with the corresponding hypotheses. These were not specified in the original protocol for the study but were planned prior to collecting the 1-year follow-up data.

**Table 3:** Comparisons related to self-reported behaviours in the 1-year follow-up

Question	Hypothesis and basis for the hypothesis
How often do you hear treatment claims?	Children in the intervention group will report hearing treatment claims more often because of being more aware of treatment claims and identifying them when they are made.
[For the last treatment claim that you heard,] did you think about what that treatment claim that you heard was based on?	A larger proportion of children in the intervention group will answer yes because of being more aware that many claims do not have a reliable basis.
How sure are you that the treatment claim you heard is true or can be trusted?	A smaller proportion of children in the intervention group will answer ‘very sure’ or ‘I don’t know’, and a larger proportion of children in the intervention group will answer this question consistently with their answer to the preceding question about the basis of the claim (Table 5) because of being better able to assess the trustworthiness of claims and many claims not having a reliable basis.
How sure are you about the advantages and disadvantages of the [most recent] treatment you used?	A higher proportion of the children in the intervention group will answer ‘not very sure because I only know about the advantages’, and a smaller proportion will answer ‘very sure’, because information about the disadvantages of treatments is often lacking. However, this difference, if there is one, will likely be small, because children in the intervention group are more likely to consider and seek information about the disadvantages of treatments.
Who do you think should decide for you whether you should use a treatment or not use a treatment?	A higher proportion of the children in the intervention group will answer that they want to be included (A, C, D, F or G) because of having learned about how to make informed health choices; and that someone who knows a lot about treatments should be included (E, F or G), because of being more aware of the importance of assessing the reliability of evidence of effects and the skills that are needed to do this. However, this difference, if there is one, will likely be small, because children in the intervention group are more likely to recognise that expert opinion alone is not a reliable basis for a claim about treatment effects.
What happens if the claim that comes in is about negative effects of the treatment?	A larger proportion of children in the intervention group will answer, ‘Not very sure because there was not a good reason behind the claims about the advantages of the treatment’, because they are more likely to identify a claim whose basis was bad.
Given your thoughts about the basis of the claim, what did you yourself decide to do about the treatment?	A smaller proportion of children in the intervention group versus the control group would choose to use a treatment (in question 29.7) having recognised that the basis of the claim was untrustworthy (in question 29.6)

Children were counted as ‘passing’ or ‘failing’ depending on whether they met a pre-specified passing score (a minimum of 13 of 24 questions answered correctly) [6]. We used a second cut-off for a score that indicated mastery of the 12 concepts (a minimum of 20 of 24 questions answered correctly).

We also report attendance and scores on national examinations for the intervention term and for the following term. These comparisons were originally planned as part of the process evaluation [13]. We asked participating schools to provide us with school attendance records and summary score sheets containing all pupils’ end-of-intervention term examination scores. The summary score sheet (Table 4) contains percentage scores for each end-of-intervention term examination and a total score across subjects (Additional file 2: Table S2). The children receive marks for English, mathematics, social studies, and science. We measured the mean difference between the intervention and control groups for each subject and for their total score (out of 100). We hypothesised higher scores in the intervention schools for English (because of the time spent reading and learning new words in English during the IHC lessons), science (based on results of randomised trials of other interventions to teach critical thinking [17–19], and stimulation of interest in science), and average scores (due to expected higher scores in English and science).

**Table 4:** Ranges of marks and points awarded for each subject

Exam score (out of 100)	Points awarded	Marks
80–100	1	Distinction 1
70–79	2	Distinction 2
65–69	3	Credit 3
60–64	4	Credit 4
55–59	5	Credit 5
50–54	6	Credit 6
45–49	7	Pass 7
35–44	8	Pass 8
Below 35	9	Failure

We asked teachers to record unexpected adverse events and problems that might pose risks to the children or others and to report these to the investigators or to the Institutional Review Board at Makerere University College of Health Sciences. Teachers in the intervention arm of the trial were given instructions for recording adverse events and problems in journals that they were asked to keep [13].

### Statistical analysis

Statistical analysis was carried out using the University of Aberdeen Health Services Research Unit’s Cluster Sample Size Calculator, applying the following assumptions: 70 children per cluster; an intraclass correlation coefficient (ICC) of 0.5, based on ICCs from a meta-analysis of randomised trials of school interventions and an international comparison of ICCs for educational achievement outcomes [20, 21]; 0% as the proportion of children expected to achieve a passing score without our intervention, based on findings from pilot testing; 10% as the smallest difference we wanted to be able to detect; an alpha of 0.05; and a power of 90%. On the basis of these assumptions, we estimated that we would need a minimum of 55 schools in each arm to detect a difference of 10% in the proportion of children with a passing score [14].

For the primary and secondary outcomes, we used mixed models with a random effects term for the clusters and the stratification variables modelled as fixed effects, using logistic regression for dichotomous outcomes and linear regression for continuous outcomes. The statistical analyses were performed with R version 3.3.2 software (R Core Team, Vienna, Austria). We used a penalized-maximum likelihood logistic regression (R package ‘logistf’) for the secondary outcome of passing scores for teachers because of rare events (only one teacher in the intervention group did not have a passing score). We converted odds ratios from logistic regression analyses to adjusted differences using the intervention group percentage as the reference. All the children and teachers who completed the test were included in the analyses.

For questions about intended behaviours and self-efficacy, we dichotomised the responses in the analysis and reported the proportions of children for each of the four response options. For comparisons of how frequently participants in both groups reported hearing treatment claims, we analysed the data as ordinal data using mixed ordinal logistic regression, and we dichotomised the responses.

User testing of the questions about self-reported behaviours by 40 children who did not participate in the trial suggested that the questions are understood by children in Uganda. In addition, we used open-ended questions to ensure that the children understood these questions correctly: ‘Please write down the treatment claim that you last heard. What did they say the treatment would change or not change about someone’s health?’ (Table 5). We coded answers to these questions as ‘correct’ or ‘incorrect’, and we excluded from the comparisons in (Table 6) all participants who did not correctly identify the type of treatment (Additional file 2: Table S3) or who did not report a treatment claim. For attendance, we compared rates in the intervention and control groups. For marks, we compared mean exam scores (Additional file 2: Table S5), the proportions of children with passing scores (≥ 35), and the proportions of children with distinction scores (≥ 70).

**Table 5:** Consistent (correct) answers regarding certainty about treatment claims^a^

If you heard about a treatment claim, what was it based on?	How sure are you that the treatment claim you heard is true or can be trusted?
Someone’s personal experience using the treatment	Not very sure, because the reason behind the claim was not good
What an expert said about it	Not very sure, because the reason behind the claim was not good
A research study that compared the treatment with another treatment or no treatment	Not very sure, because the reason behind the claim was not good OR Very sure, because the reason behind the claim was good
Something else	Not very sure, because the reason behind the claim was not good
I could not tell what the treatment claim was based on	Not very sure, because I don’t know the reason behind the claim

^a^Questions 28.5 and 28.6 in Additional file 1

**Table 6:** Exclusion criteria for self-reported behaviours

Response options for questions 28.2 and 29.3	Response to questions 28.3 and 29.4
28.2 What was the treatment in the claim you last heard about?	28.3 Please write the claim that you last heard.
29.3 What was the treatment for which you or an adult made the decision?	What was the claim about the treatment for which you or an adult made the decision?
Using a medicine (e.g., taking a tablet or syrup)	Exclude if the claim is not about a medicine
Getting an operation (e.g., removing a bad tooth)	Exclude if the claim is not about an operation
Using something to feel better or to heal more quickly (e.g., using a bandage or glasses)	Exclude if the claim is not about equipment
Something else (eating food or drinking something to feel better; e.g., herbs or fruit)	Exclude if the claim is not about eating/drinking something (e.g., herbs or fruit)
Avoiding doing something to feel better (e.g., not drinking milk)	Exclude if the claim is not about avoiding something
Something else	Exclude if the claim is not about a treatment (‘anything done to care for yourself, so you stay well or, if you are sick or injured, so you get better and not worse’)

To explore the risk of bias due to attrition, which was larger in the control schools than in the intervention schools, we conducted two sensitivity analyses. First, we conducted an analysis using inverse probability weighting. In this, the children in each school were given a weight equal to the inverse of the proportion of children in the school who had completed the test. Second, using the Lee bounds approach [22], we calculated upper and lower bounds for the mean difference in test scores. The bounds are constructed by trimming the group with less attrition at the upper and lower tails of the outcome (test score) distribution, respectively. In this analysis, the sample was trimmed in the intervention schools so that the proportion of children included in the analysis was equal for both groups. We did not adjust for covariates in the sensitivity analysis.

We conducted two additional sensitivity analyses to explore why the effects for the primary outcomes were smaller after 1 year than they were at the end of the intervention term. First, we calculated the differences between effects (adjusted mean differences and odds ratios) between the first and second tests based on mixed models with a random effects term for the clusters (schools) and individuals (who are used twice in these analyses), and the stratification variables modelled as fixed effects, using linear regression for the mean scores and logistic regression for the proportions with a passing score. Second, we estimated the effects at the end of the intervention term, excluding children who did not take the second test, using the same model as described above.

We explored whether there were differences in the effect of the intervention for children with advanced reading skills (all four literacy questions answered correctly) versus basic reading skills (both basic literacy questions correct and one or two of the advanced literacy questions wrong) versus lacking basic reading skills (one or both basic literacy questions wrong). In order to put the effect of the intervention in the context of effect sizes reported for other interventions to improve critical thinking or learning in primary schools [23], we calculated the adjusted standardised mean difference (Hedges’ *g*) and its 95% confidence interval using formulae described by White and Thomas [24].

Parents of 675 children in either the intervention or control group were recruited to participate in a parallel trial [16]. That trial evaluated the effects of a podcast designed to teach the parents of primary school children nine IHC key concepts, eight of which were included in the primary school resources. We conducted a second subgroup analysis to explore whether having a parent who listened to the podcast improved the scores of the children and whether there was an interaction between the effect of the podcast and the primary school resources. Because the parents allocated to listen to the podcast did not do so until after the children had completed the tests the first time, we only conducted this analysis for the 1-year follow-up study. We used statistical models as described above for this analysis; the main effects of the podcast were also included in these analyses.

## Results

All 120 schools that were randomised provided data for the primary outcome measures and were included in the primary analyses. Most of the schools in both groups were urban or semi-urban (Table 7). There were more public schools in the control group (55% versus 50%). For the 1-year follow-up, there were fewer teachers who taught science as their main subject. Teachers in Ugandan primary schools frequently move and switch the major subject that they teach due to changes in staffing. Therefore, changes in the main subject taught by teachers are not unusual. There were otherwise only minor differences in the characteristics of the participants between the end of the intervention term and the 1-year follow-up, and between the intervention and control groups.

**Table 7:** Characteristics of the participants

		One-year follow-up		End of intervention term	
		Control schools	Intervention schools	Control schools	Intervention schools
Schools (selected from the central region of Uganda)		*N* = 60	*N* = 60	*N* = 60	*N* = 60
Location	Rural	8 (13%)	6 (10%)	8 (13%)	6 (10%)
	Semi-urban	15 (25%)	14 (23%)	15 (25%)	14 (23%)
	Urban	37 (62%)	40 (67%)	37 (62%)	40 (67%)
Ownership	Public	33 (55%)	30 (50%)	33 (55%)	30 (50%)
	Private	27 (45%)	30 (50%)	27 (45%)	30 (50%)
Teachers^a^		*N* = 74	*N* = 85	*N* = 74	*N* = 85
Completed tests		59 (80%)	78 (92%)	67 (91%)	85 (100%)
Education	Certificate	27 (46%)	34 (44%)	30 (45%)	39 (46%)
	Diploma	31 (53%)	35 (45%)	33 (49%)	35 (41%)
	University degree	1 (2%)	9 (12%)	3 (4%)	10 (12%)
Main subject taught	Science	32 (54%)	48 (56%)	49 (73%)	68 (80%)
Sex	Women	24 (41%)	32 (45%)	29 (43%)	34 (40%)
Children (enrolled in year-5 at the start of the term)		*N* = 6256	*N* = 6383	*N* = 6256	*N* = 6383
Completed tests^b^		2844 (45%)	3943 (62%)	4430 (71%)	5753 (90%)
Completed tests per school^c^	Median (25th to 75th percentile) (Range)	40 (24 to 57) (4 to 300)	49 (30 to 77) (10 to 270)	60 (40 to 95) (12 to 150)	61 (43 to 89) (18 to 176)
Sex	Girls	1558 (55%)	2164 (55%)	2457 (55%)	3154 (55%)
Age	Median (25th to 75th percentile) (Range)	12 (10 to 14) (9 to 18)	12 (10 to 14) (8 to 19)	11 (10 to 12) (8 to 20)	11 (10 to 12) (8 to 18)

^a^The number of teachers who completed the test at the end of the first term. Head teachers were initially asked to identify teacher who taught science to children in the fifth year of primary school. However, some schools had more than one year 5 class. Six intervention schools with more than one year 5 class (with a different teacher for each class) requested that nine additional teachers be included altogether

^b^Questions about the characteristics of the teachers and children were included in the test completed at the end of the school term and 1 year later

^c^The average number of year 5 children per school at the start of the term was 84 in both groups

Six intervention schools had more than one year 5 class (with a different teacher for each class). This resulted in nine more teachers receiving training and being included in the intervention schools. No teachers were added in the control schools, because the teachers in the control schools did not receive training. For the 1-year follow-up, 78 (92%) of the teachers in the intervention schools and 59 (88%) of the teachers in the control schools completed the same test that the children took at the end of the term.

Altogether, 6787 children completed the 1-year follow-up test (Table 7). As was the case with the test taken at the end of the intervention term, more children completed the follow-up test in the intervention schools (62%) than in the control schools (45%). We think this is because teachers who taught the lessons were more motivated to arrange for the children whom they had taught to take the test. The proportion of girls (55%) and the median age of children (12 years; 25th to 75th percentile, 10 to 14) in the two groups were the same. Most of the children answered all the questions. The proportion of missing values (unanswered questions) for each question was between 0.25% and 3.38%, and the number of missing values was similar between the intervention and control schools (Additional file 2: Table S4).

Only 64 schools provided data on the secondary outcome of school attendance. Ninety-three schools provided data on examination scores for the intervention term, and 99 provided data for the following term (Additional file 2: Table S5).

### Primary outcomes and sensitivity analyses

The average score for children in the intervention schools was 68.7% compared with 53.0% in the control schools (Table 8). The adjusted mean difference (based on the regression analysis) was 16.7% (95% CI, 13.9% to 19.5%; *P* < 0.00001) higher in the intervention than in the control group. The distribution of test scores is shown in Additional file 3. In the intervention schools, 80.1% of the children had a passing score (≥ 13 of 24 correct answers) compared with 51.5% in the control schools (Table 8). The adjusted difference (based on the odds ratio from the logistic regression analysis) was 39.5% more children who passed (95% CI, 29.9% to 47.5%) in the intervention group than in the control group. Although the average score and the proportion of children with a passing score in the intervention group were higher after 1 year than at the end of the intervention term, the differences between the intervention and control schools were smaller, because the scores increased more in the control schools than in the intervention schools between the first and second tests.

**Table 8:** Main test score results at 1-year follow-up

	Control schools	Intervention schools	Adjusted difference^a^	Odds ratio^a^	ICC
Primary outcome					
One-year follow-up					
Mean score, %	Mean score: 53.0% (SD 16.8%)	Mean score: 68.7% (SD 18.2%)	Mean difference: 16.7% (95% CI, 13.9% to 19.5%) *P* < 0.00001		0.18
End of intervention term					
Mean score, %	Mean score: 43.1% (SD 15.2%)	Mean score: 62.4% (SD 18.8%)	Mean difference: 20.0% (95% CI, 17.3% to 22.7%)		0.18
One-year follow-up					
Passing score (≥ 13 of 24 correct answers)	51.5% of children (*n* = 1464/2844)	80.1% of children (*n* = 3160/3943)	39.5% more children (95% CI, 29.9% to 47.5%)	5.88 (95% CI, 4.00 to 8.33) *P* < 0.00001	0.20
End of intervention term					
Passing score (≥ 13 of 24 correct answers)	26.8% of children (*n* = 1186/4430)	69.0% of children (*n* = 3967/5753)	49.8% more children (95% CI, 43.8% to 54.6%)	9.34 (95% CI, 6.62 to 13.18)	0.19
Secondary outcomes					
One-year follow-up					
Mastery score (≥ 20 of 24 correct answers)	4.9% of children (*n* = 139/2844)	28.9% of children (*n* = 1138/3943)	25.0% more children (23.2–26.5%)	10.00 (95% CI, 6.67 to 16.67) *P* < 0.00001	0.19
End of intervention term					
Mastery score (≥ 20 of 24 correct answers)	0.9% of children (*n* = 38/4430)	18.6% of children (*n* = 1070/5753)	18.0% more children (95% CI, 17.5% to 18.2%)	35.33 (95% CI, 20.58 to 60.67)	0.21
Teachers’ scores					
One-year follow-up					
Mean score, %	Mean score: 68.5% (SD 14.9%)	Mean score: 86.2% (SD 10.2%)	Mean difference: 17.5% (13.2% to 21.8%) *P* < 0.00001		
End of intervention term					
Mean score, %	Mean score: 66.7% (SD 14.3%)	Mean score: 84.6% (SD 17.1%)	Mean difference: 18.3% (95% CI, 12.9% to 23.3%)		
One-year follow-up					
Passing score (≥ 13 of 24 correct answers)	85.9% of teachers (*n* = 50/59)	98.7% of teachers (*n* = 77/78)	9.4% more teachers (1.3% to 52.0%)	9.12^b^ (95% CI, 2.01 to 86.7) *P* = 0.003	
End of intervention term					
Passing score (≥ 13 of 24 correct answers)	86.6% of teachers (*n* = 58/67)	97.6% of teachers (*n* = 83/85)	11.3% more teachers (95% CI, 4.0% to 13.0%)	7.24 (95% CI, 1.49 to 35.26)	
One-year follow-up					
Mastery score (≥ 20 of 24 correct answers)	22.0% of teachers (*n* = 13/59)	67.9% of teachers (*n* = 53/78)	46.5% more teachers (28.1% to 61.3%)	7.70 (95% CI, 3.56 to 17.70) *P* < 0.00001	
End of intervention term					
Mastery score (≥ 20 of 24 correct answers)	14.9% of teachers (*n* = 10/67)	71.8% of teachers (*n* = 61/85)	56.7% more teachers (95% CI, 37.3% to 70.4%)	14.38 (95% CI, 6.24 to 33.14)	

^a^The adjusted difference is based on mixed models with a random effects term for the clusters (for the children only) and the stratification variables modelled as fixed effects, using logistic regression for dichotomous outcomes and linear regression for continuous outcomes. The odds ratios from the logistic regressions have been converted to differences based on the intervention school proportions and the odds ratios calculated using the intervention schools as the reference (the inverse of the odds ratios shown here)

^b^Penalized-maximum likelihood logistic regression (R package ‘logistf’) was used for this analysis because of rare events (only one teacher in the intervention group did not have a passing score)

We conducted two sensitivity analyses to investigate possible explanations for the small effect estimates after 1 year. To explore whether the apparent differences might have occurred by chance alone, we calculated the probability of a difference as large as or larger than what we observed having occurred by chance (Additional file 2: Table S18). It is highly unlikely that the differences in the effect estimates would have occurred by chance (*P* > 0.00001). To explore whether the differences might reflect bias resulting from differential loss to follow-up, we calculated the effects at the end of the intervention term, excluding children who did not take the second test (Additional file 2: Table S19). The effect estimates are similar. We consider other possible explanations in the ‘Discussion’ section below.

We conducted two sensitivity analyses to assess the potential risk of bias from attrition (i.e., children who did not take the test) (Table 9). There was very little difference between the results of analysis using inverse probability weighting and the primary analysis (Additional file 2: Table S6), suggesting that the results are robust. In the second analysis, we calculated Lee bounds for the mean difference in test scores. This resulted in lower (worst case) and upper (best case) mean differences of 6.4% and 26.6%, respectively (95% CI, 6.6% to 26.5%). This indicates that even with the worst-case scenario, the average test score in the intervention schools was still 6.4% higher than in the control schools. Moreover, the worst-case scenario, which removed 17% of the children with the highest test scores from the intervention group, is unlikely. This is equivalent to assuming that the children in the control schools who did not take the test would have had scores that corresponded to the top 17% of the children in the intervention schools, had they taken the test (Additional file 2: Table S7). It is more likely that the children who were lost to follow-up and did not take the test would have done worse rather than better than the children who did take the test.

**Table 9:** Sensitivity analyses at 1-year follow-up

	Adjusted difference^a^	Odds ratio
Mean score		
Primary analysis	Mean difference: 16.7% (95% CI, 13.9% to 19.5%) *P* < 0.00001	
Weighted analysis	Mean difference: 16.7% (95% CI, 13.9% to 19.5%)	
Lee bounds	6.4% to 26.6% (95% CI, 6.6% to 26.5%)	
Passing score (≥ 13 of 24 correct answers)		
Primary analysis	39.5% (95% CI, 29.9% to 47.5%)	5.88 (95% CI, 4.00 to 8.33) *P* < 0.0001
Weighted analysis	40.9% (95% CI, 31.0% to 49.4%)	6.25 (95% CI, 4.17 to 9.09) *P* < 0.0001

^a^The adjusted difference is based on mixed models with a random effects term for the clusters and the stratification variables modelled as fixed effects, using logistic regression for dichotomous outcomes and linear regression for continuous outcomes. The odds ratios from the logistic regressions for passing scores have been converted to differences based on the intervention school proportions and the odds ratios calculated using the intervention schools as the reference (the inverse of the odds ratios shown here)

### Secondary outcomes

- *The proportion of children with a score indicating mastery of the concepts*

In the intervention schools, 28.9% of the children had a score indicating mastery of the 12 key concepts (≥ 20 of 24 correct answers) compared with 4.9% of the children in the control schools (Table 8). The adjusted difference was 25.0% more children in the intervention schools who mastered the concepts (95% CI, 23.2% to 26.5%). This is a larger difference than there was at the end of the term during which the intervention had been delivered (18.0%). The proportion of children with a score indicating mastery increased from 18.6% to 28.9% in the intervention group between the first and second tests, compared with an increase from 0.9% to 4.9% in the control group.

- *For each concept, the proportion of children who answered both questions correctly*

For each concept, the proportion of children who answered both questions correctly was higher in the intervention schools than in the control schools, including for the concept that was not covered in the primary school resources (*P* < 0.0001 for all 13 concepts after a Bonferroni correction for multiple comparisons) (Table 10).

- *Children’s intended behaviours and self-efficacy*

**Table 10:** Results for each concept for children at 1-year follow-up

No.	Concept	Control schools % correct^a^ No. of schools = 60 No. of children = 2844	Intervention schools % correct^a^ No. of schools = 60 No. of children = 3943	Adjusted difference^b^ (95% CI)	ICC^c^	Odds ratio (95% CI)
	Claims					
1.1	Treatments may be harmful.	40.5% (*n* = 1152)	64.6% (*n* = 2547)	29.2% (22.4–35.0%)	0.120	3.33 (2.50–4.35) *P* < 0.00001
1.2	Personal experiences or anecdotes (stories) are an unreliable basis for assessing the effects of most treatments.	26.5% (*n* = 753)	52.0% (*n* = 2052)	30.0% (24.5–34.2%)	0.119	3.85 (2.86–5.00) *P* < 0.00001
1.3	A treatment outcome may be associated with a treatment, but not caused by the treatment.^d^	27.3% (*n* = 776)	36.4% (*n* = 1436)	11.2% (6.4–15.2%)	0.087	1.69 (1.33–2.13) *P* = 0.00002
1.4	Widely used treatments or treatments that have been used for a long time are not necessarily beneficial or safe.	26,3% (*n* = 748)	54,4% (*n* = 2144)	30.0% (23.8–35.1%)	0,157	3.70 (2.70–5.00) *P* < 0.00001
1.5	New, brand-named, or more expensive treatments may not be better than available alternatives.	48.9% (*n* = 1392)	73.6% (*n* = 2901)	28.1% (22.2–34.5%)	0.088	3.33 (2.63–4.35) *P* < 0.00001
1.6	Opinions of experts or authorities do not alone provide a reliable basis for deciding on the benefits and harms of treatments.	43.2% (*n* = 1230)	67.6% (*n* = 2664)	26.8% (20.3–33.3%)	0.113	3.03 (2.33–4.00) *P* < 0.00001
1.7	Conflicting interests may result in misleading claims about the effects of treatments.	37.0% (*n* = 1051)	47.2% (*n* = 1861)	10.8% (5.5–15.9%)	0.077	1.56 (1.25–1.96) 0.00009
	Comparisons					
2.1	Evaluating the effects of treatments requires appropriate comparisons.	10.3% (*n* = 294)	32.0% (*n* = 1263)	24.2% (21.1–26.2%)	0.148	5.56 (3.85–7.69) *P* < 0.00001
2.2	A part from the treatments being compared, the comparison groups need to be similar (i.e., ‘like needs to be compared with like’).	12.1% (*n* = 344)	29.3% (*n* = 1155)	16.6% (14.2–18.9%)	0.063	2.86 (2.33–3.57) *P* < 0.00001
2.5	If possible, people should not know which of the treatments being compared they are receiving.	23.3% (*n* = 664)	36.2% (*n* = 1428)	15.1% (11.4–18.8%)	0.070	2.13 (1.72–2.70) *P* < 0.00001
3.1	Small studies in which few outcome events occur are usually not informative and the results may be misleading.	32.6% (*n* = 928)	50.3% (*n* = 1984)	20.5% (15.8–25.3%)	0.082	2.38 (1.92–3.03) *P* < 0.00001
4.1	The results of single comparisons of treatments can be misleading.	29.1% (*n* = 827)	44.8% (*n* = 1766)	17.6% (12.4–22.2%)	0.096	2.17 (1.69–2.78) *P* < 0.00001
	Choices					
5.1	Treatments usually have beneficial and harmful effects.	35.2% (*n* = 1000)	50.8% (*n* = 2004)	16.8% (11.4–22.1%)	0.090	2.00 (1.59–2.56) *P* < 0.00001

^a^There were two multiple-choice questions for each concept. The proportions are for the percentage of children who answered both questions correctly

^b^The adjusted difference is based on mixed models with a random effects term for the clusters and the stratification variables modelled as fixed effects, using logistic regression. The odds ratios from the logistic regressions have been converted to differences based on the intervention school proportions and the inverse of the odds ratios shown here

^c^Intraclass correlation coefficient

^d^This concept was not included in the learning resources or counted in the average, pass or mastery scores

Compared with children in the control schools, children in the intervention schools were more likely to respond that they would find out the basis for a claim (adjusted difference, 8.1%; 95% CI, 3.7% to 12.6%) and to participate in a research study if asked (adjusted difference, 7.7%; 95% CI, 2.0% to 13.5%) (Additional file 2: Table S8). These findings are similar to those we found 1 year earlier. However, there was little if any difference in how likely they were to find out if a claim was based on research (adjusted difference, 2.6%; 95% CI, − 1.9% to 7.2%). This contrasts with what we found 1 year earlier (10.8%; 95% CI, 6.3% to 15.1%).

- *Self-reported behaviours*

Similar to what we found 1 year earlier, children in the intervention schools were more likely to consider it easy to assess whether a claim is based on research than children in the control schools (adjusted difference, 14.8%; 95% CI, 8.9% to 20.5%) (Table 11). They were also more likely to consider it easy to find information about treatments based on research (adjusted difference, 7.2%; 95% CI, 2.6% to 11.5%) (Table 12), whereas 1 year earlier, we had detected little if any difference (Additional file 2: Table S9). We detected little if any difference in how easy children thought it was to assess how sure they could be about the results of research or to assess how relevant research findings are to them. One year earlier, compared with children in the control group, the children in the intervention group were less likely to consider it easy to assess how sure they could be about the results of research.

**Table 11:** Intended behaviours at 1-year follow-up

Think about an illness that you might get. Imagine someone claiming (saying) that a particular treatment might help you get better.						
	How likely are you to find out what the claim was based on (e.g., by asking the person making the claim)?		How likely are you to find out if the claim was based on a research study comparing the treatment with no treatment (a fair comparison)?		How likely are you to say ‘yes’ if you are asked to participate in a research study comparing two treatments for your illness (a fair comparison)?	
	Control schools *N* = 2844	Intervention schools *N* = 3943	Control schools *N* = 2844	Intervention schools *N* = 3943	Control schools *N* = 2844	Intervention schools *N* = 3943
Missing	69 (2.4%)	67 (1.7%)	87 (3.1%)	70 (1.8%)	36 (1.3%)	44 (1.1%)
Very unlikely	217 (7.6%)	376 (9.5%)	301 (10.6%)	467 (11.8%)	245 (8.6%)	277 (7.0%)
Unlikely	289 (10.2%)	376 (9.5%)	424 (14.9%)	569 (14.4%)	329 (11.6%)	429 (10.9%)
Likely	975 (34.3%)	1510 (38.3%)	747 (26.3%)	997 (25.3%)	1045 (36.7%)	1577 (40.0%)
Very likely	678 (23.8%)	1082 (27.4%)	705 (24.8%)	1164 (29.5%)	719 (25.3%)	1155 (29.3%)
I don’t know	616 (21.7%)	532 (13.5%)	580 (20.4%)	676 (17.1%)	470 (16.5%)	461 (11.7%)
Likely or very likely^a^	1653 (58.1%)	2592 (65.7%)	1452 (51.1%)	2161 (54.8%)	1764 (62.0%)	2732 (69.3%)
Odds ratio (95% CI)^b^	1.41 (1.18–1.69) *P* = 0.00020		1.11 (0.93–1.33) *P* = 0.269		1.41 (1.10–1.79) *P* = 0.00629	
Adjusted difference^b^	8.1% (3.7–12.6%)		2.6% (−1.9% to 7.2%)		7.7% (2.0–13.5%)	
End of intervention term^c^						
Likely or very likely	2440 (55.1%)	3731 (64.9%)	1967 (44.4%)	3114 (54.1%)	2163 (48.8%)	3201 (55.6%)
Odds ratio	1.56 (95% CI, 1.29 to 1.88)		1.54 (95% CI, 1.29 to 1.84)		1.37 (95% CI, 1.16 to 1.62)	
Adjusted difference	10.6% (95% CI, 6.2% to 14.7%)		10.8% (95% CI, 6.3% to 15.1%)		7.8% (95% CI, 3.7% to 11.9%)	

^a^ Missing values and don’t know are pooled with unlikely and very unlikely

^b^The difference is an adjusted difference, based on mixed models with a random effects term for the clusters and the stratification variables modelled as fixed effects, using logistic regression. The odds ratios from the logistic regressions have been converted to differences using the intervention schools as the reference and the inverse of the odds ratios shown here

^c^Results based on responses at the end of the term when the intervention was delivered

**Table 12:** Self-efficacy

How difficult or easy would you find each of these actions to be?								
	Assessing whether a claim about a treatment is based on a research study comparing treatments (a fair comparison)		Assessing where I can find information about treatments that is based on research studies comparing treatments (fair comparisons)		Assessing how sure I can be about the results of a research study comparing treatments (the trustworthiness of the results)		Assessing if the results of a research study comparing treatments are likely to be relevant to me	
	Control schools *N* = 2844	Intervention schools *N* = 3943	Control schools *N* = 2844	Intervention schools *N* = 3943	Control schools *N* = 2844	Intervention schools *N* = 3943	Control schools *N* = 2844	Intervention schools *N* = 3943
Missing	71 (2.5%)	55 (1.4%)	73 (2.6%)	71 (1.8%)	82 (2.9%)	84 (2.1%)	72 (2.5%)	86 (2.2%)
Very difficult	357 (12.6%)	455 (11.5%)	338 (11.9%)	431 (10.9%)	488 (17.2%)	581 (14.7%)	436 (15.3%)	568 (14.4%)
Difficult	779 (27.4%)	865 (21.9%)	634 (22.3%)	876 (22.2%)	653 (23.0%)	1007 (25.5%)	513 (18.0%)	727 (18.4%)
Easy	837 (29.4%)	1517 (38.5%)	899 (31.6%)	1348 (34.2%)	640 (22.5%)	897 (22.7%)	694 (24.4%)	1027 (26.0%)
Very easy	334 (11.7%)	623 (15.8%)	525 (18.5%)	856 (21.7%)	454 (16.0%)	712 (18.1%)	562 (19.8%)	779 (19.8%)
I don’t know	466 (16.4%)	428 (10.9%)	375 (13.2%)	361 (9.2%)	527 (18.5%)	662 (16.8%)	567 (19.9%)	756 (19.2%)
Easy or svery easy^a^	1171 (41.2%)	2140 (54.3%)	1424 (50.1%)	2204 (55.9%)	1094 (38.5%)	1609 (40.8%)	1256 (44.2%)	1806 (45.8%)
Odds ratio (95% CI)^b^	1.82 (1.43–2.33) *P* < 0.00001		1.33 (1.11–1.59) *P* = 0.00171		1.10 (0.94–1.30) *P* = 0.233		1.10 (0.93–1.28) *P* = 0.279	
Adjusted difference^b^	14.8% (8.9–20.5%)		7.2% (2.6–11.5%)		2.3% (− 1.4% to 6.1%)		2.3% (− 1.9% to 6.1%)	
End of intervention term^c^								
Easy or very easy	1886 (42.6%)	3244 (56.4%)	3069 (53.3%)	2238 (50.5%)	1777 (40.1%)	2112 (36.7%)	2002 (45.2%)	2727 (47.4%)
Odds ratio	1.83 (95% CI, 1.55 to 2.16)		1.13 (95% CI, 0.96 to 1.33)		0.84 (95% CI, 0.73 to 0.96)		1.08 (95% CI, 0.93 to 1.25)	
Adjusted difference	15.0% (95% CI, 10.9% to 19.0%)		3.0% (95% CI, − 1.0% to 7.0%)		− 4.1% (95% CI, − 1.0% to − 7.3%)		1.9% (95% CI, − 1.8% to 5.6%)	

^a^Missing values and don’t know are pooled with difficult and very difficult

^b^The difference is an adjusted difference, based on mixed models with a random effects term for the clusters and the stratification variables modelled as fixed effects, using logistic regression. The odds ratios from the logistic regressions have been converted to differences using the intervention schools as the reference and the inverse of the odds ratios shown here

^c^Results based on responses at the end of the term when the intervention was delivered

The children in the intervention schools were more likely to report hearing one or more treatment claims daily or weekly (Table 13) than were children in the control schools (adjusted difference, 7.0%; 95% CI, 0.5% to 12.9%) (Additional file 2: Table S10). The children in the intervention schools were less likely to be very sure or not to know whether a claim could be trusted (Table 14) (adjusted difference, − 15%; 95% CI, − 9.9% to − 19.7%) and more likely to assess the trustworthiness of a claim consistently with what they identified as the basis of the claim (adjusted difference, 7.6%; 95% CI, 3.5% to 11.1%) (Additional file 2: Table S11). However, there were only slight differences in how likely children in the intervention schools were to think about the basis of the last claim that they heard (Table 15) (adjusted difference, 4.1%; 95% CI, − 1.2% to 9.6%) (Additional file 2: Table S12 and S13), as well as in their assessments of the advantages and disadvantages of the most recent treatment they had used (Table 16) (Additional file 2: Table S14). The difference in attendance or examination scores was also small (Additional file 2: Table S5). As reported previously [14], none of the teachers or research assistants who observed the lessons reported any adverse events.

- *Mean, passing and mastery scores for teachers*

**Table 13:** Self-reported behaviour: awareness of treatment claims

How often do you hear treatment claims?		
	Control schools *N* = 2844	Intervention schools *N* = 3943
One or more most days	572 (20.1%)	1000 (25.4%)
One or more most weeks	374 (13.2%)	599 (15.2%)
One or more most months	497 (17.5%)	715 (18.1%)
Almost never	653 (23.0%)	788 (20.0%)
I don’t know	717 (25.2%)	810 (20.5%)
Missing	31 (1.1%)	31 (0.8%)
One or more most days or most weeks	946 (33.8%)	1599 (40.6%)
Odds ratio^a^	1.35 (95% CI, 1.02–1.79) *P* = 0.0356	
Adjusted difference^b^	7.0% (95% CI, 0.5–12.9%)	

^a^The odds ratio for the dichotomised data is shown in the table. The odds ratio from the mixed ordinal logistic regression was 1.30 (95% CI, 1.01 to 1.67; *P* = 0.0431)

^b^The difference is an adjusted difference, based on a mixed model with a random effects term for the clusters and the stratification variables modelled as fixed effects, using logistic regression. The odds ratio from the logistic regression has been converted to a difference using the intervention schools as the reference and the inverse of the odds ratios shown here

**Table 14:** Self-reported behaviour: assessment of trustworthiness of treatment claims

How sure are you that the treatment claim you heard is true or can be trusted?		
	Control schools *N* = 2844	Intervention schools *N* = 3943
Missing	49 (1.7%)	60 (1.5%)
Not very sure because I don’t know the reason behind the claim	665 (23.4%)	1039 (26.4%)
Not very sure because the reason behind the claim was not good	543 (19.1%)	1087 (27.6%)
Very sure because the reason behind the claim was good	704 (24.8%)	790 (20.0%)
I don’t know because I don’t know how to decide whether it is true or not	883 (31.0%)	967 (24.5%)
Very sure or I don’t know	1587 (55.8%)	1757 (44.6%)
Odds ratio (very sure or I don’t know vs other)	0.55 (95% CI, 0.45–0.67) *P* < 0.0001	
Adjusted difference^a^	−15.0% (95% CI, − 9.9% to − 19.7%)	
Odds ratio (consistent with what they identified as the basis for the claim)^b^	1.45 (95% CI, 1.18–1.75) *P* = 0.000549	
Adjusted difference^a^	7.6% (95% CI 3.5% - 11.1%)	

^a^The differences are adjusted differences, based on mixed models with a random effects term for the clusters and the stratification variables modelled as fixed effects, using logistic regression. The odds ratio from the logistic regression has been converted to a difference using the intervention schools as the reference and the inverse of the odds ratios shown here

^b^See Table 5

**Table 15:** Self-reported behaviour: assessment of the basis of treatment claims

For the last treatment claim that you heard, did you think about what that treatment claim that you heard was based on?		
	Control schools *N* = 2844	Intervention schools *N* = 3943
Missing	50 (1.8%)	57 (1.4%)
No	512 (18.0%)	845 (21.4%)
Yes	1387 (48.8%)	2116 (53.7%)
I don’t remember	895 (31.5%)	925 (23.5%)
Odds ratio (yes versus other)	1.18 (95% CI, 0.95–1.47) *P* = 0.130	
Adjusted difference^a^	4.1% (95% CI, −1.2% to 9.6%)	

^a^The difference is an adjusted difference, based on a mixed model with a random effects term for the clusters and the stratification variables modelled as fixed effects, using logistic regression. The odds ratio from the logistic regression has been converted to a difference using the intervention schools as the reference and the inverse of the odds ratios shown here

**Table 16:** Self-reported behaviour: assessment of advantages and disadvantages of treatments

How sure are you about the advantages and disadvantages of the [most recent] treatment you used?		
	Control schools *N* = 2844	Intervention schools *N* = 3943
A. Not very sure because I don’t know the reasons behind the claims about the good and bad things that treatment makes happen	531 (18.7%)	851 (21.6%)
B. Not very sure because there was not a good reason behind the claims about the advantages of the treatment	355 (12.5%)	549 (13.9%)
C. Not very sure because I only know about the advantages of the treatment. I also need to know about the disadvantages	765 (26.9%)	992 (25.2%)
D. Very sure because there is a good reason behind the claims about the advantages and disadvantages of the treatment	652 (22.9%)	929 (23.6%)
E. I did not use any treatment	498 (17.5%)	590 (15.0%)
Missing	43 (1.5%)	32 (0.8%)
Odds ratio (C versus any other response)	1.05 (95% CI, 0.86–1.30) *P* = 0.62	
Adjusted difference answer C vs else	−0.9% (95% CI, −5.3% to 2.7%)	
Odds ratio (D versus any other response)	1.03 (95% CI, 0.85–1.23) *P* = 0.79	
Adjusted difference answer D vs else	−0.5% (95% CI, −3.9% to 2.8%)	

After 1 year, most teachers in both the intervention and control groups (98.7% and 85.9%, respectively) had passing scores (adjusted difference, 8.6%; 95% CI, 1% to 55.5%) (Table 8). The teachers in the intervention group were much more likely to have a score indicating mastery of the concepts (67.9% versus 21.9%; adjusted difference, 46.3%; 95% CI, 31.5% to 56.6%). These results are similar to those we found at the end of the intervention term.

### Subgroup analyses

As was the case at the end of the intervention term, the intervention still had positive effects 1 year later, regardless of reading skills (Table 17), but with larger effects for children with better reading skills (Additional file 2: Table S15). Compared with the control schools (Table 18), reading skills were better in the intervention schools at the end of the intervention term and after 1 year (Additional file 2: Table S16). They had improved by about the same amount in both the intervention and control schools after 1 year. We did not detect an interaction between having a parent who listened to the podcast and the primary school intervention (Table 19) (adjusted difference for the interaction, 3.8%; 95% CI, − 3.9% to 11.4%) (Additional file 2: Table S17).

**Table 17:** Subgroup analysis: reading skills^a^

	Control schools	Intervention schools	Adjusted difference^b^	Odds ratio	ICC
Mean score, %					
Lacking basic reading skills (*N* = 1775)	No. of children = 893	No. of children = 882			
	Mean score: 47.2% (SD 16.4%)	Mean score: 57.1% (SD 18.1%)	Mean difference: 11.2% (95% CI, 8.2% to 14.2%)		0.146
Basic reading skills (*N* = 2672)	No. of children = 1093	No. of children = 1579			
	Mean score: 55.2% (SD 16.9%)	Mean score: 67.9% (SD 16.8%)	Mean difference: 14.8% (95% CI, 12.3% to 17.3%)		0.162
Advanced reading skills (*N* = 2340)	No. of children = 858	No. of children = 1482			
	Mean score: 56.3% (SD 15.6%)	Mean score: 76.5% (SD 15.5%)	Mean difference: 19.4% (95% CI, 16.9% to 21.9%)		0.117
Passing score (≥ 13 of 24 correct answers)					
Lacking basic reading skills (*N* = 1775)	No. of children = 893	No. of children = 882			
	36.6% of children *n* = 327	59.3% of children *n* = 523	28.9% more children (95% CI, 20.8% to 36.7%)	0.30 (95% CI, 0.20 to 0.43)	0.144
Basic reading skills (*N* = 2672)	No. of children = 1093	No. of children = 1579			
	57.0% of children *n* = 623	81.2% of children *n* = 1282	33.6% more children (95% CI, 24.0% to 41.9%)	0.21 (95% CI, 0.15 to 0.31)	0.150
Advanced reading skills (*N* = 2340)	No. of children = 858	No. of children = 1482			
	60.0% of children *n* = 514	91.4% of children *n* = 1355	33.4% more children (95% CI, 25.7% to 42.5%)	0.13 (95% CI, 0.09 to 0.18)	0.098
Mastery score (≥ 20 of 24 correct answers)					
Lacking basic reading skills (*N* = 1775)	No. of children = 893	No. of children = 882		0.22	
	3.0% of children *n* = 27	10,1% of children *n* = 89	7.7% more children (95% CI, 5.6% to 8.8%)	(95% CI, 0.12 to 0.42)	0.220
Basic reading skills (*n* = 2672)	No. of children = 1093	No. of children = 1579		0.15	
	6.5% of children *n* = 71	24.1% of children *n* = 380	19.6% more children (95% CI, 17.0% to 21.3%)	(95% CI, 0.09 to 0.24)	0.192
Advanced reading skills (*n* = 2340)	No. of children = 858	No. of children = 1482		0.06	
	4.8% of children *n* = 41	45.1% of children *n* = 669	40.4% more children (95% CI, 38.2% to 41.9%)	(95% CI, 0.04 to 0.09)	0.139

^a^Because reading skills were measured after the intervention, we have not reported a test of interaction here (see Additional file 2)

^b^The adjusted difference is based on mixed models with a random effects term for the clusters and the stratification variables modelled as fixed effects, using logistic regression for dichotomous outcomes and linear regression for continuous outcomes. The odds ratios from the logistic regressions for passing scores and mastery scores have been converted to differences using the intervention school proportions and the inverse of the odds ratios shown here

**Table 18:** Differences in reading skills

Reading skills	Immediately after the intervention^a^			One-year follow-up^a^			Change from first to second test^a^		
	Control schools No. of children 4412 *n* (%)	Intervention schools No. of children 5711 *n* (%)	Diff	Control schools No. of children 2844 *n* (%)	Intervention schools No. of children 3943 *n* (%)	Diff	Control schools	Intervention schools	Diff
Lacking basic reading skills	2139 (48.5%)	2224 (38.9%)	−9.5%	893 (31.4%)	882 (22.4%)	−9.0%	−17.1%	−16.6%	0.5%
Basic reading skills	1507 (34.2%)	2155 37.7%	3.6%	1093 (38.4%)	1579 (40.0%)	1.6%	4.3%	2.3%	−2.0%
Advanced reading skills	766 (17.4%)	1332 23.3%	6.0%	858 (30.2%)	1482 (37.6%)	7.4%	12.8%	14.3%	1.5%

^a^Reading skills as measured by first four questions in the test administered at the end of the term when the intervention was delivered and the same test 1 year later. The differences (Diff) are shown between the intervention and control schools for each time the test was administered and the change from the first to the second time

**Table 19:** Subgroup analysis: parent who listened to the podcast

	Control schools	Intervention schools	Adjusted effect of the interaction^a^
	No. of children = 69	No. of children = 98	Mean difference: 3.8% (95% CI, − 3.9% to 11.4%) *P* = 0.3443
Parent in control group (*N* = 167)	Mean score: 55.1% (SD 16.4%)	Mean score: 64.5% (SD 20.2%)	
	No. of children = 64	No. of children = 104	
Parent in podcast group (*N* = 168)	Mean score: 53.6% (SD 15.9%)	Mean score: 66.3% (SD 18.6%)	

^a^Adjusted for location, ownership (public/private) and random effect of clustering, ICC = 0.185

## Discussion

The large effect that the Informed Health Choices intervention had on the ability of primary school children in Uganda to assess claims about treatment effects was sustained after 1 year. The mean score and the proportions of children with passing and mastery scores increased in the intervention schools (Table 8). However, because the scores in the control schools increased more than the scores in the intervention schools, the differences between the intervention and control schools for the mean score and the proportion of children with a passing score were smaller, albeit still large. On the other hand, the difference in the proportion of children with a mastery score increased.

We considered five possible explanations for these findings, none of which seem likely. First, the apparent differences in the effect estimates between the first and second measurements is unlikely to have occurred by chance alone (Additional file 2: Table S18). Second, bias resulting from differential loss to follow-up is also unlikely to explain the differences (Additional file 2: Table S19). A third possible explanation is that there was a learning effect from taking the test the first time, which was greater in the control schools than in the intervention schools. It is possible that the learning effect of taking the test alone would be greater than the added learning effect of taking the test after having been exposed to the IHC lessons. ‘Testing effects’—gains in learning that occur when students take a practice test—are well documented [25, 26]. They occur with and without feedback [26] and for higher-level thinking (‘application’ in Bloom’s taxonomy) as well as for recall of basic facts [25]. However, most studies investigating testing effects have been conducted over a much shorter time frame [26], and we are not aware of any studies that have documented a difference in testing effects between students who studied before taking a practice test and others who did not study. A fourth possible explanation is that children learn to think critically about treatment claims naturally as they grow older or through the existing curriculum, and the control schools were catching up with the intervention schools because of this. However, as documented in our process evaluation, the content of the lessons was new for all of the teachers and not something that they had previously taught. Furthermore, we did not deliver the learning resources to the control schools until after the follow-up data had been collected. Fifth, it also seems unlikely that the improvement was due to an improvement in reading skills in the control schools, because the change in reading skills was similar in the intervention and control schools.

The effects that we found for the children for each IHC key concept, as well as the effects that we found for the teachers, were similar to those we found at the end of the intervention term. Overall, these findings support the conclusion that the effects of the intervention were sustained, even though we are unable to explain why the children’s scores increased more in the control schools than in the intervention schools.

Other findings provide modest support for the conclusion that the children in the intervention schools were more likely to use what they had learned. The children in the intervention schools remained more likely than those in control schools to find out the basis for a treatment claim, more confident in their ability to assess whether a treatment claim is based on research, and more likely to participate in a research study if asked. They also appeared to be somewhat more aware of treatment claims, more sceptical of treatment claims, and more likely to assess the trustworthiness of treatment claims. However, all of these differences were smaller than the difference for the primary outcome measures. Moreover, at the end of the intervention term, children in the intervention schools were more likely than children in the control schools to say they would find out if a treatment claim was based on research, but after 1 year there was little difference.

The data we were able to collect for attendance and national examinations were incomplete, but based on those data, there was little difference between children in the intervention and control schools (Table 20). This contrasts with findings of studies in the United Kingdom, which have shown beneficial effects of critical thinking or meta-cognition interventions on academic achievement [17–19]. Possible explanations for this include the limitations of the data we were able to collect for these outcomes and differences between the interventions and the contexts in which they were delivered.

**Table 20:** Attendance and national examinations

Attendance rates				
	Control schools *N* = 33 schools Median (25th to 75th percentile)	Intervention schools *N* = 31 schools Median (25th to 75th percentile)	Adjusted difference	*P* value
Intervention term	90.3% (78.7% to 98.0%)	89.1% (80.4% to 96.4%)	3% less (95% CI, −14 to 6)	0.437
Following term	91.7% (81.1% to 97.8%)	89.5% (78.6% to 96.2%)	2% more (95% CI, −10 to 13)	0.726
Average scores on national examinations				
	Control schools Mean (SD)	Intervention schools Mean (SD)	Adjusted mean difference	*P* value
End of intervention term				
English	54.2% (22.5)	52.3% (22.5)	−1.7% (95% CI, −6.6 to 3.2)	0.500
Math	51.5% (23.4)	49.0% (22.5)	−1.8% (95% CI, −6.6 to 3.0)	0.457
Science	49.8% (24.4)	49.7% (23.3)	−0.5% (95% CI, −5.4 to 4.5)	0.852
Social science	52.6% (24.0)	51.9% (23.7)	−1.0% (95% CI, −6.2 to 4.2)	0.699
Total	52.3% (21.4)	51.1% (21.0)	−1.2% (95% CI, − 5.5 to 3.2)	0.597
Following term				
English	56.3% (22.1)	56.1% (22.5)	2.4% (95% CI, −2.3 to 7.2)	0.312
Math	53.8% (23.2)	50.2% (22.4)	0.8% (95% CI, −4.1 to 5.8)	0.752
Science	52.4% (23.9)	49.3% (23.3)	0.8% (95% CI, − 4.1 to 5.4)	0.813
Social science	56.0% (23.8)	52.0% (22.7)	−0.1% (95% CI, −4.8 to 4.7)	0.964
Total	54.8% (21.5)	52.2% (20.6)	1.0% (95% CI, −3.4, 5.4)	0.671
Proportion with a passing score (≥ 35%) on the national examinations				
	Control schools *n* (%)	Intervention schools *n* (%)	Adjusted difference	
End of intervention term	Total: 49 schools, 3795 children	Total: 44 schools, 4201 children		
English	2917/3766 (77.5%)	3009/3984 (71.8%)	0.0% (95% CI, −10.0 to 13.8)	0.998
Math	2709/3772 (71.8%)	2809/3985 (70.5%)	1.6% (95% CI, −12.0 to 11.9)	0.799
Science	2632/3764 (69.9%)	2829/3990 (70.9%)	−0.1% (95% CI, −11.4 to 14.6)	0.988
Social science	2794/3773 (74.1%)	2957/3980 (74.3%)	−1.7% (95% CI, − 11.9 to 12.9)	0.801
Total	2698/3730 (72.3%)	2830/3934 (71.9%)	−0.7% (95% CI, − 11.5 to 13.8)	0.920
Following term	Total: 51 schools, 3956 children	Total: 48 schools, 4474 children		
English	3205/3934 (81.5%)	3655/4460 (82.0%)	3.8% (95% CI, −5.2 to 16.6)	0.461
Math	3038/3940 (76.9%)	3174/4441 (71.5%)	−0.1% (95% CI, −10.3 to 12.8)	0.984
Science	2923/3942 (74.2%)	3137/4436 (70.7%)	−0.1% (95% CI, −11.4 to 14.6)	0.878
Social science	3125/3940 (79.3%)	3366/4452 (75.6%)	1.1 (95% CI, −8.1 to 13.2)	0.839
Total	3022/3914 (77.2%)	3268/4404 (74.2%)	1.5% (95% CI, −8.6 to 14.8)	0.797
Proportion with a distinction score (≥ 70%) on the national examinations				
	Control schools *n* (%)	Intervention schools *n* (%)	Adjusted difference	
End of intervention term	Total: 49 schools, 3795 children	Total: 44 schools, 4201 children		
English	1133/3766 (30.1%)	1077/3984 (27.0%)	−7.0% (95% CI, −21.4 to 4.9)	0.278
Math	995/3772 (26.4%)	850/3985 (21.3%)	−4.2% (95% CI, −17.3 to 5.6)	0.716
Science	966/3764 (25.7%)	977/3990 (24.5%)	−2.1% (95% CI, −14.9 to 7.7)	0.716
Social science	1117/3773 (29.6%)	1117/3980 (28.1%)	−1.7% (95% CI, − 15.5 to 9.2)	0.791
Total	904/3730 (24.2%)	882/3934 (22.4%)	−2.1% (95% CI, − 15.0 to 7.3)	0.693
Following term	Total: 51 schools, 3956 children	Total: 48 schools, 4474 children		
English	1263/3934 (32.1%)	1440/4460 (32.3%)	4.8% (95% CI, −7.7 to 14.6)	0.425
Math	1101/3940 (27.9%)	1023/4441 (23.0%)	−3.4% (95% CI, −16.8 to 6.6)	0.551
Science	1099/3942 (27.9%)	1024/4436 (23.1%)	−0.8% (95% CI, −12.3 to 7.9)	0.875
Social science	1342/3940 (34.1%)	1207/4452 (27.1%)	−0.2% (95% CI, − 12.4 to 9.3)	0.967
Total	1063 (27.2%)	1012 (23.0%)	1.3% (95% CI, −11.1 to 10.0)	0.819

*SD* standard deviation

The main limitations of our follow-up study are similar to those discussed in our report of effects found immediately after the intervention [14]. First, we cannot rule out some degree of bias due to attrition. However, sensitivity analyses suggest that the effect estimates are robust. Second, we used an outcome measure that we developed ourselves. Outcome measures developed by the study authors for use in a study may be more likely to find larger effects than studies using established measures of critical thinking [23]. We developed the outcome measure because there was no pre-existing outcome measure suitable for our study [8]. Although we have demonstrated the validity and reliability of the outcome measure [6, 7, 9, 10], one should be cautious about comparing our results with the effects of other critical thinking interventions. Moreover, we are unaware of any other directly comparable studies [20, 23, 27–30]. Other interventions in primary schools have been found to improve critical thinking [23], but these studies have been conducted in high-income countries, few have measured outcomes after 1 year, and neither the interventions nor the outcome measures are directly comparable [27, 29].

It remains uncertain how transferable the findings of this study are to other countries. However, pilot testing in Kenya, Norway and Rwanda suggest that it may be possible to use the IHC primary school resources without substantial modifications. They have already been translated to Kiswahili, Kinyarwanda, Spanish, French and Farsi. There are plans or expressions of interest to translate them to other languages, including Chinese, German and Italian. Pilot studies have been completed or planned in several other countries, including Ireland and South Africa. The resources are open access, and we have prepared a guide for translating, contextualising and testing them [31].

However, we believe that a one-off intervention is unlikely to have large long-term effects on decision-making, health behaviours or health. Rather, we view this as the first step in developing a set of interventions for a spiral curriculum [32, 33]. Using this approach, some of the IHC key concepts would be introduced, as we did in this study. Then those concepts would be reinforced in subsequent cycles, and other, more complex concepts would be introduced.

## Conclusions

It is possible to teach young children in a low-income country to think critically about the trustworthiness of claims about the benefits and harms of treatments, and children retain what they have learned for at least 1 year. In this study, we were also able to document modest effects on self-reported behaviours, because young children seldom make actual health choices independently. We believe it is highly desirable to begin teaching the IHC key concepts at a young age, and we have shown that this is possible.

## Supplementary information

**Additional file 1.** The claim evaluation tools.

**Additional file 2: Table S1.** Comparisons related to self-reported behaviours in the 1-year follow-up. **Table S2.** Ranges of marks and points awarded for each subject. **Table S3.** Exclusion criteria for self-reported behaviours. **Table S4.** Number of missing values for each question. **Table S5.** Attendance and national examinations. **Table S6.** Sensitivity analyses – 1-year follow-up. **Table S7.** Attrition, differences in test scores across strata of schools. **Table S8.** Intended behaviours – 1-year follow-up. **Table S9.** Self-efficacy. **Table S10.** Self-reported behaviour – awareness of treatment claims. **Table S11.** Self-reported behaviour – assessment of trustworthiness of treatment claims. **Table S12.** Consistent (correct) answers regarding certainty about treatment claims. **Table S13.** Self-reported behaviour – assessment of the basis of treatment claims. **Table S14.** Self-reported behaviour – assessment of advantages and disadvantages of treatments. **Table S15.** Subgroup analysis – reading skills. **Table S16.** Differences in reading skills. **Table S17.** Subgroup analysis – parent who listened to the podcast. **Table S18.** Exploratory analyses – *P* values for differences between first (end of intervention term) and second (1-year follow-up) effects. **Table S19.** Exploratory analyses excluding children who did not take the test both times.

**Additional file 3.** Distribution of scores and curves.

## Data Availability

The data files for the 1-year follow-up are available from the Norwegian Centre for Research Data (http://www.nsd.uib.no/nsd/english/index.html).

## Abbreviations

IHC: Informed Health Choices Project
