# Supplementary material for: Effects of the Informed Health Choices primary school intervention on the ability of children in Uganda to assess the reliability of claims about treatment effects, 1-year follow-up: a cluster-randomised trial
Source: Trials. 2020 Jan 6;21:27. doi: 10.1186/s13063-019-3960-9 (PMC6945419; doi:10.1186/s13063-019-3960-9)
Supplement: Supplementary file 2 — Additional file 2: Table S1. Comparisons related to self-reported behaviours in the 1-year follow-up. Table S2. Ranges of marks and points awarded for each subject. Table S3. Exclusion criteria for self-reported behaviours. Table S4. Number of missing values for each question. Table S5. Attendance and national examinations. Table S6. Sensitivity analyses – 1-year follow-up. Table S7. Attrition, differences in test scores across strata of schools. Table S8. Intended behaviours – 1-year follow-up. Table S9. Self-efficacy. Table S10. Self-reported behaviour – awareness of treatment claims. Table S11. Self-reported behaviour – assessment of trustworthiness of treatment claims. Table S12. Consistent (correct) answers regarding certainty about treatment claims. Table S13. Self-reported behaviour – assessment of the basis of treatment claims. Table S14. Self-reported behaviour – assessment of advantages and disadvantages of treatments. Table S15. Subgroup analysis – reading skills. Table S16. Differences in reading skills. Table S17. Subgroup analysis – parent who listened to the podcast. Table S18. Exploratory analyses – P values for differences between first (end of intervention term) and second (1-year follow-up) effects. Table S19. Exploratory analyses excluding children who did not take the test both times. [file 13063_2019_3960_MOESM2_ESM.docx]

**Additional file 2. Additional tables**

Table S1. Comparisons related to self-reported behaviours in the one-year follow-up

Table S2. Ranges of marks and points awarded for each subject

Table S3. Exclusion criteria for self-reported behaviours

Table S4. Number of missing values for each question

Table S5. Attendance and national examinations

Table S6. Sensitivity analyses - one-year follow-up

Table S7. Attrition, differences in test scores across strata of schools

Table S8. Intended behaviours - one-year follow-up

Table S9. Self-efficacy

Table S10. Self-reported behaviour - awareness of treatment claims

Table S11. Self-reported behaviour - assessment of trustworthiness of treatment claims

Table S12. Consistent (correct) answers regarding certainty about treatment claims

Table S13. Self-reported behaviour - assessment of the basis of treatment claims

Table S14. Self-reported behaviour - assessment of advantages and disadvantages of treatments

Table S15. Subgroup analysis - reading skills

Table S16. Differences in reading skills

Table S17. Subgroup analysis - parent who listened to the podcast

Table S18. Exploratory analyses - p-values for differences between first (end of intervention term) and second (one-year follow-up) effects

Table S19. Exploratory analyses excluding children who did not take the test both times

**Table S1. Comparisons related to self-reported behaviours in the one-year follow-up**

| **Question** | **Hypothesis and basis for the hypothesis** |
| --- | --- |
| How often do you hear treatment claims? | Children in the intervention group will report hearing treatment claims more often because of being more aware of treatment claims and identifying them when they are made. |
| [For the last treatment claim that you heard,] did you think about what that treatment claim that you heard was based on? | A larger proportion of children in the intervention group will answer yes because of being more aware that many claims do not have a reliable basis. |
| How sure are you that the treatment claim you heard is true or can be trusted? | A smaller proportion of children in the intervention group will answer “very sure” or “I don’t know”, and a larger proportion of children in the intervention group will answer this question consistently with their answer to the preceding question about the basis of the claim (Table 3) because of being better able to assess the trustworthiness of claims and many claims not having a reliable basis. |
| How sure are you about the advantages and disadvantages of the [most recent] treatment you used? | A higher proportion of the children in the intervention group will answer “not very sure because I only know about the advantages” and a smaller proportion will answer “very sure”, because information about the disadvantages of treatments is often lacking. However, this difference, if there is one, will likely be small, because children in the intervention group are more likely to consider and seek information about the disadvantages of treatments. |
| Who do you think should decide for you whether you should use a treatment or not use a treatment? | A higher proportion of the children in the intervention group will answer that they want to be included (A, C, D, F, or G) because of having learned about how to make informed health choices; and that someone who knows a lot about treatments should be included (E, F, or G), because of being more aware of the importance of assessing the reliability of evidence of effects and the skills that are needed to do this. However, this difference, if there is one, will likely be small, because children in the intervention group are more likely to recognise that expert opinion alone is not a reliable basis for a claim about treatment effects. What happens if the claim that comes in is about negative effects of the treatment?  A larger proportion of children in the intervention group will answer, “Not very sure because there was not a good reason behind the claims about the advantages of the treatment”, because they are more likely to identify a claim whose basis was bad. |
| Given your thoughts about the basis of the claim, what did you yourself decide to do about the treatment? | A smaller proportion of children in the intervention group versus the control group would choose to use a treatment (in question 29.7) having recognised that the basis of the claim was untrustworthy (in question 29.6) |

**Table S2. Ranges of marks and points awarded for each subject**

| **Exam score**  (out of 100) | **Points**  **awarded** | **Marks** |
| --- | --- | --- |
| 80-100 | 1 | Distinction 1 |
| 70-79 | 2 | Distinction 2 |
| 65-69 | 3 | Credit 3 |
| 60-64 | 4 | Credit 4 |
| 55-59 | 5 | Credit 5 |
| 50-54 | 6 | Credit 6 |
| 45-49 | 7 | Pass 7 |
| 35-44 | 8 | Pass 8 |
| Below 35 | 9 | Failure |

**Table S3. Exclusion criteria for self-reported behaviours**

| **Response options for questions 28.2 and 29.3** | **Response to questions 28.3 and 29.4** |
| --- | --- |
| 28.2 What was the treatment in the claim you last heard about | 28.3 Please write the claim that you last heard |
| 29.3 What was the treatment for which you or an adult made the decision? | What was the claim about the treatment for which you or an adult made the decision? |
| Using a medicine (e.g. taking a tablet or syrup) | Exclude if the claim is not about a medicine |
| Getting an operation (e.g. removing a bad tooth) | Exclude if the claim is not about an operation |
| Using something to feel better or to heal more quickly (e.g. using a bandage or glasses) | Exclude if the claim is not about equipment |
| Something else (Eating food or drinking something to feel better (e.g. herbs or fruit)) | Exclude if the claim is not about eating/drinking something e.g. herbs or fruit |
| Avoiding doing something to feel better (e.g. not drinking milk) | Exclude if the claim is not about avoiding something |
| Something else | Exclude if the claim is not about a treatment (“anything done to care for yourself, so you stay well or, if you are sick or injured, so you get better and not worse”) |

**Table S4. Number of missing values for each question**

|  | **Number of unanswered questions** | | | |
| --- | --- | --- | --- | --- |
|  | **Control**  (n=2844) | | **Intervention**  (n=3943) | |
|  | **N** | **%** | **N** | **%** |
| Question 4 | 9 | 0.32% | 20 | 0.51% |
| Question 5 | 8 | 0.28% | 26 | 0.66% |
| Question 6 | 8 | 0.28% | 17 | 0.43% |
| Question 7 | 8 | 0.28% | 16 | 0.41% |
| Question 8 | 8 | 0.28% | 25 | 0.63% |
| Question 9 | 10 | 0.35% | 16 | 0.41% |
| Question 10 | 3 | 0.11% | 13 | 0.33% |
| Question 11 | 7 | 0.25% | 7 | 0.18% |
| Question 12 | 12 | 0.42% | 21 | 0.53% |
| Question 13 | 8 | 0.28% | 19 | 0.48% |
| Question 14 | 24 | 0.84% | 38 | 0.96% |
| Question 15 | 22 | 0.77% | 39 | 0.99% |
| Question 16 | 19 | 0.67% | 30 | 0.76% |
| Question 17 | 31 | 1.09% | 35 | 0.89% |
| Question 18 | 21 | 0.74% | 16 | 0.41% |
| Question 19 | 19 | 0.67% | 16 | 0.41% |
| Question 20 | 32 | 1.13% | 19 | 0.48% |
| Question 21 part 1 | 79 | 2.78% | 39 | 0.99% |
| Question 21 part 2 | 96 | 3.38% | 54 | 1.37% |
| Question 21 part 3 | 95 | 3.34% | 53 | 1.34% |
| Question 21 part 4 | 87 | 3.06% | 43 | 1.09% |
| Question 22 part 1 | 79 | 2.78% | 50 | 1.27% |
| Question 22 part 2 | 59 | 2.07% | 54 | 1.37% |
| Question 22 part 3 | 93 | 3.27% | 70 | 1.78% |
| Question 23 part 1 | 69 | 2.43% | 53 | 1.34% |
| Question 23 part 2 | 52 | 1.83% | 44 | 1.12% |
| Question 24 part 1 | 69 | 2.43% | 67 | 1.70% |
| Question 24 part 2 | 87 | 3.06% | 70 | 1.78% |
| Question 25 part 1 | 71 | 2.50% | 55 | 1.39% |
| Question 25 part 2 | 73 | 2.57% | 71 | 1.80% |
| Question 25 part 3 | 82 | 2.88% | 84 | 2.13% |
| Question 25 part 4 | 72 | 2.53% | 86 | 2.18% |

**Table S5. Attendance and national examinations**

| **Attendance rates** | | | | |
| --- | --- | --- | --- | --- |
|  | **Control schools**  N=33 schools  Median (25^th^ to 75^th^ percentile) | **Intervention schools**  N=31 schools  Median (25th to 75th percentile) | **Adjusted difference** | **P-value** |
| **Intervention term** | 90.3% (78.7% to 98.0%) | 89.1% (80.4% to 96.4%) | 3% less (95% CI -14 to 6) | 0.437 |
| **Following term** | 91.7% (81.1% to 97.8%) | 89.5% (78.6% to 96.2%) | 2% more (95% CI -10 to 13) | 0.726 |
| **Average scores on national examinations** | | | | |
|  | **Control schools**  Mean (SD) | **Intervention schools**  Mean (SD) | **Adjusted mean difference** | **P-value** |
| **End of intervention term** |  |  |  |  |
| English | 54.2% (22.5) | 52.3% (22.5) | -1.7% (95% CI -6.6 to 3.2) | 0.500 |
| Math | 51.5% (23.4) | 49.0% (22.5) | -1.8% (95% CI -6.6 to 3.0) | 0.457 |
| Science | 49.8% (24.4) | 49.7% (23.3) | -0.5% (95% CI -5.4 to 4.5) | 0.852 |
| Social science | 52.6% (24.0) | 51.9% (23.7) | -1.0% (95% CI -6.2 to 4.2) | 0.699 |
| Total | 52.3% (21.4) | 51.1% (21.0) | -1.2% (-5.5 to 3.2) | 0.597 |
| **Following term** |  |  |  |  |
| English | 56.3% (22.1) | 56.1% (22.5) | 2.4% (95% CI -2.3 to 7.2) | 0.312 |
| Math | 53.8% (23.2) | 50.2% (22.4) | 0.8% (95% CI -4.1 to 5.8) | 0.752 |
| Science | 52.4% (23.9) | 49.3% (23.3) | 0.8% (95% CI -4.1 to 5.4) | 0.813 |
| Social science | 56.0% (23.8) | 52.0% (22.7) | -0.1% (95% CI -4.8 to 4.7) | 0.964 |
| Total | 54.8% (21.5) | 52.2 % (20.6) | 1.0% (-3.4, 5.4) | 0.671 |
| **Proportion with a passing score (> 35%) on the national examinations** | | | | |
|  | **Control schools**  N (%) | **Intervention schools**  N (%) | **Adjusted difference** |  |
| **End of intervention term** | Total: 49 schools, 3795 children | Total: 44 schools, 4201 children |  |  |
| English | 2917/3766 (77.5%) | 3009/3984 (71.8%) | 0.0% (95% CI -10.0 to 13.8) | 0.998 |
| Math | 2709/3772 (71.8%) | 2809/3985 (70.5%) | 1.6% (95% CI -12.0 to 11.9) | 0.799 |
| Science | 2632/3764 (69.9%) | 2829/3990 (70.9%) | -0.1% (95% CI -11.4 to 14.6) | 0.988 |
| Social science | 2794/3773 (74.1%) | 2957/3980 (74.3%) | -1.7% (95% CI -11.9 to 12.9) | 0.801 |
| Total | 2698/3730 (72.3%) | 2830/3934 (71.9%) | -0.7% (95% CI -11.5 to 13.8) | 0.920 |
| **Following term** | Total: 51 schools, 3956 children | Total: 48 schools, 4474 children |  |  |
| English | 3205/3934 (81.5%) | 3655/4460 (82.0%) | 3.8% (95% CI -5.2 to 16.6) | 0.461 |
| Math | 3038/3940 (76.9%) | 3174/4441 (71.5%) | -0.1% (95% CI -10.3 to 12.8) | 0.984 |
| Science | 2923/3942 (74.2%) | 3137/4436 (70.7%) | -0.1% (95% CI -11.4 to 14.6) | 0.878 |
| Social science | 3125/3940 (79.3%) | 3366/4452 (75.6%) | 1.1 (95% CI -8.1 to 13.2) | 0.839 |
| Total | 3022/3914 (77.2%) | 3268/4404 (74.2%) | 1.5% (95% CI -8.6 to 14.8) | 0.797 |
| **Proportion with a distinction score (> 70%) on the national examinations** | | | | |
|  | **Control schools**  N (%) | **Intervention schools**  N (%) | **Adjusted difference** |  |
| **End of intervention term** | Total: 49 schools, 3795 children | Total: 44 schools, 4201 children |  |  |
| English | 1133/3766 (30.1%) | 1077/3984 (27.0%) | -7.0% (95% CI -21.4 to 4.9) | 0.278 |
| Math | 995/3772 (26.4%) | 850/3985 (21.3%) | -4.2% (95% CI -17.3 to 5.6) | 0.716 |
| Science | 966/3764 (25.7%) | 977/3990 (24.5%) | -2.1% (95% CI -14.9 to 7.7) | 0.716 |
| Social science | 1117/3773 (29.6%) | 1117/3980 (28.1%) | -1.7% (95% CI -15.5 to 9.2) | 0.791 |
| Total | 904/3730 (24.2%) | 882/3934 (22.4%) | -2.1% (95% CI-15.0 to 7.3) | 0.693 |
| **Following term** | Total: 51 schools, 3956 children | Total: 48 schools, 4474 children |  |  |
| English | 1263/3934 (32.1%) | 1440/4460 (32.3%) | 4.8% (95% CI -7.7 to 14.6) | 0.425 |
| Math | 1101/3940 (27.9%) | 1023/4441 (23.0%) | -3.4% (95% CI -16.8 to 6.6) | 0.551 |
| Science | 1099/3942 (27.9%) | 1024/4436 (23.1%) | -0.8% (95% CI -12.3 to 7.9) | 0.875 |
| Social science | 1342/3940 (34.1%) | 1207/4452 (27.1%) | -0.2% (95% CI -12.4 to 9.3) | 0.967 |
| Total | 1063 (27.2%) | 1012 (23.0%) | 1.3% (95%CI -11.1 to 10.0) | 0.819 |

SD = standard deviation

**Table S6. Sensitivity analyses - one-year follow-up**

|  | **Adjusted difference*** | **Odds ratio** |
| --- | --- | --- |
| **Mean score** |  |  |
| Primary analysis | **Mean difference: 16.7%**  (95% CI 13.9% to 19.5%)  P <0.00001 |  |
| Weighted analysis | **Mean difference:** **16.7%**  (95% CI 13.9% to 19.5%) |  |
| Lee bounds | **6.4% to 26.6%**  (95% CI 6.6% to 26.5%) |  |
| **Passing score**  (> 13 out of 24 correct answers) |  |  |
| Primary analysis | **39.5%**  (95% CI 29.9%-47.5%) | **5.88**  (95% CI 4.00 to 8.33)  P<0.0001 |
| Weighted analysis | **40.9%**  (95% CI 31.0% to 49.4%) | **6.25**  (95% CI 4.17 to 9.09)  P<0.0001 |

* The adjusted difference is based on mixed models with a random effects term for the clusters and the stratification variables modelled as fixed effects, using logistic regression for dichotomous outcomes and linear regression for continuous outcomes. The odds ratios from the logistic regressions for passing scores have been converted to differences based on the intervention school proportions and the odds ratios calculated using the intervention schools as the reference (the inverse of the odds ratios shown here).

**Table S7. Attrition, differences in test scores across strata of schools**

**Proportion of children who completed the test**

| **Control schools** | |  |  |  |
| --- | --- | --- | --- | --- |
|  | **Government** | **Private** | **Total** | **N (%) of schools** |
| Rural | 35.1% (124/353) | 43.7% (62/142) | 37.6% (186/495) | 8 (13.3%) |
| Semi-urban | 40.8% (430/1055) | 53.7% (116/216) | 43% (546/1271) | 15 (25%) |
| Urban | 43.1% (957/2219) | 50.9% (1155/2271) | 47% (2112/4490) | 37 (61.7%) |
| **Total** | **41.7% (1511/3627)** | **50.7% (1333/2629)** | **45.5% (2844/6256)** | **60 (100%)** |
|  |  |  |  |  |
| **Intervention schools** | |  |  |  |
|  | **Government** | **Private** | **Total** | **N (%) of schools** |
| Rural | 42.1% (117/278) | 60.6% (175/289) | 51.5% (292/567) | 6 (10%) |
| Semi-urban | 59.6% (766/1286) | 75.7% (467/617) | 64.8% (1233/1903) | 14 (23.3%) |
| Urban | 60.6% (1406/2319) | 63.5% (1012/1594) | 61.8% (2418/3913) | 40 (66.7%) |
| **Total** | **58.9% (2289/3883)** | **66.2% (1654/2500)** | **61.8% (3943/6383)** | **60 (100%)** |

**Test scores**

|  | Treatment effect | School ownership effect | School location effect |
| --- | --- | --- | --- |
| **Mean score** |  |  |  |
| Without weighting | 16.7%  (95% CI 13.9 to 19.5)  P < 0.00001 | 7.2%  (95% CI 4.3 to 10.0)  P < 0.00001 | 0.2%  (95% CI -1.9 to 2.3)  P = 0.872 |
| Weighted | 16.7%  (95% CI 13.9 to 19.5)  P < 0.00001 | 7.2%  (95% CI: 4.3 to 10.1)  P < 0.00001 | 0.3%  (95% CI -1.8to 2.3)  P = 0.807 |
| **Passing score** (> 13 out of 24 correct answers) | | | |
| Without weighting | OR 0.17  (95% CI 0.12 to 0.25)  P < 0.00001 | OR 2.14  (95% CI 1.49 to 3.09)  P = 0.00004 | OR 0.99  (95% CI 0.76 to 1.29)  P = 0.92 |
| Weighted | OR 0.16  (95% CI 0.11 to 0.24)  P < 0.00001 | OR 2.28  (95% CI 1.54 to 3.38)  P = 0.00004 | OR 1.00  (95% CI 0.75 to 1.32)  P = 0.99 |
| **Mastery score** (> 20 out of 24 correct answers) | | | |
| Without weighting | OR 0.10  (95% CI 0.06 to 0.15) P < 0.00001 | OR 2.34  (95% CI 1.59 to 3.46)  P = 0.00002 | OR 0.99  (95% CI 0.74 to 1.33)  P = 0.951 |
| Weighted | OR 0.09  (95% CI 0.06 to 0.13) P < 0.00001 | OR 2.59  (95% CI 1.72 to 3.90)  P = 0.0005 | OR 1.06  (95% CI 0.78 to 1.44)  P = 0.712 |

OR: odds ratio

**Table S8. Intended behaviours - one-year follow-up**

*Think about an illness that you might get. Imagine someone claiming (saying) that a particular treatment might help you get better.*

|  | **How likely are you to find out what the claim was based on (for example by asking the person making the claim)?** | | **How likely are you to find out if the claim was based on a research study comparing the treatment to no treatment (a fair comparison)?** | | **How likely are you to say “yes” if you are asked to participate in a research study comparing two treatments for your illness (a fair comparison)?** | |
| --- | --- | --- | --- | --- | --- | --- |
|  | **Control schools**  N=2844 | **Intervention schools**  N=3943 | **Control schools**  N=2844 | **Intervention schools**  N=3943 | **Control schools**  N= 2844 | **Intervention schools**  N= 3943 |
| Missing | 69 (2.4%) | 67 (1.7%) | 87 (3.1%) | 70 (1.8%) | 36 (1.3%) | 44 (1.1%) |
| Very unlikely | 217 (7.6%) | 376 (9.5%) | 301 (10.6%) | 467 (11.8%) | 245 (8.6%) | 277 (7.0%) |
| Unlikely | 289 (10.2%) | 376 (9.5%) | 424 (14.9%) | 569 (14.4%) | 329 (11.6%) | 429 (10.9%) |
| Likely | 975 (34.3%) | 1510 (38.3%) | 747 (26.3%) | 997 (25.3%) | 1045 (36.7%) | 1577 (40.0%) |
| Very likely | 678 (23.8%) | 1082 (27.4%) | 705 (24.8%) | 1164 (29.5%) | 719 (25.3%) | 1155 (29.3%) |
| I don’t know | 616 (21.7%) | 532 (13.5%) | 580 (20.4%) | 676 (17.1%) | 470 (16.5%) | 461 (11.7%) |
| **Likely or**  **very likely*** | **1653 (58.1%)** | **2592 (65.7%)** | **1452 (51.1%)** | **2161 (54.8%)** | **1764 (62.0%)** | **2732 (69.3%)** |
| **Odds ratio (95% CI)^†^** | 1.41  (1.18 - 1.69)  P=0.00020 | | 1.11  (0.93 - 1.33 )  P=0.269 | | 1.41  (1.10 - 1.79)  P=0.00629 | |
| **Adjusted Difference^†^** | **8.1%**  (3.7%-12.6%) | | **2.6%**  (-1.9% - 7.2%) | | **7.7%**  (2.0% - 13.5%) | |
| **End of intervention term^‡^** | | | | | | |

| **Likely or**  **very likely** | **2440 (55.1%)** | **3731 (64.9%)** | **1967 (44.4%)** | **3114 (54.1%)** | **2163 (48.8%)** | **3201 (55.6%)** |
| --- | --- | --- | --- | --- | --- | --- |
| **Odds ratio** | 1.56  (95% CI 1.29 to 1.88) | | 1.54  (95% CI 1.29 to 1.84) | | 1.37  (95% CI 1.16 to 1.62) | |
| **Adjusted Difference** | **10.6%**  (95% CI 6.2% to 14.7%) | | **10.8%**  (95% CI 6.3% to 15.1%) | | **7.8%**  (95% CI 3.7% to 11.9%) | |

* Missing values and don’t know are pooled with unlikely and very unlikely.

^†^ The difference is an adjusted difference, based on mixed models with a random effects term for the clusters and the stratification variables modelled as fixed effects, using logistic regression. The odds ratios from the logistic regressions have been converted to differences using the intervention schools as the reference and the inverse of the odds ratios shown here.

^‡^ Results based on responses at the end of the term when the intervention was delivered.

**Table S9. Self-efficacy**

*How difficult or easy would you find each of these actions to be?*

|  | **Assessing whether a claim about a treatment is based on a research study comparing treatments (a fair comparison)** | | **Assessing where I can find information about treatments that is based on research studies comparing treatments (fair comparisons)** | | **Assessing how sure I can be about the results of a research study comparing treatments (the trustworthiness of the results)** | | **Assessing if the results of a research study comparing treatments are likely to be relevant to me** | |
| --- | --- | --- | --- | --- | --- | --- | --- | --- |
|  | **Control schools**  N=2844 | **Intervention schools**  N=3943 | **Control schools**  N=2844 | **Intervention schools**  N=3943 | **Control schools**  N=2844 | **Intervention schools**  N=3943 | **Control schools**  N=2844 | **Intervention schools**  N=3943 |
| Missing | 71  (2.5%) | 55  (1.4%) | 73  (2.6%) | 71  (1.8%) | 82  (2.9%) | 84  (2.1%) | 72  (2.5%) | 86  (2.2%) |
| Very difficult | 357 (12.6%) | 455 (11.5%) | 338 (11.9%) | 431 (10.9%) | 488 (17.2%) | 581 (14.7%) | 436 (15.3%) | 568 (14.4%) |
| Difficult | 779 (27.4%) | 865 (21.9%) | 634 (22.3%) | 876 (22.2%) | 653 (23.0%) | 1007 (25.5%) | 513 (18.0%) | 727 (18.4%) |
| Easy | 837 (29.4%) | 1517 (38.5%) | 899 (31.6%) | 1348 (34.2%) | 640 (22.5%) | 897 (22.7%) | 694 (24.4%) | 1027 (26.0%) |
| Very easy | 334 (11.7%) | 623 (15.8%) | 525 (18.5%) | 856 (21.7%) | 454 (16.0%) | 712 (18.1%) | 562 (19.8%) | 779 (19.8%) |
| I don’t know | 466 (16.4%) | 428 (10.9%) | 375 (13.2%) | 361 (9.2%) | 527 (18.5%) | 662 (16.8%) | 567 (19.9%) | 756 (19.2%) |
| **Easy or**  **very easy*** | **1171 (41.2%)** | **2140 (54.3%)** | **1424 (50.1%)** | **2204 (55.9%)** | **1094 (38.5%)** | **1609 (40.8%)** | **1256 (44.2%)** | **1806 (45.8%)** |
| **Odds ratio (95% CI)^†^** | 1.82  (1.43 - 2.33 )  P<0.00001 | | 1.33  (1.11 - 1.59)  P=0.00171 | | 1.10  (0.94 - 1.30)  P=0.233 | | 1.10  (0.93 - 1.28)  P=0.279 | |
| **Adjusted difference^†^** | **14.8%**  (8.9% - 20.5%) | | **7.2%**  (2.6% – 11.5%) | | **2.3%**  (-1.4% - 6.1%) | | **2.3%**  (-1.9% - 6.1%) | |
| **End of intervention term^‡^** | | | | | | | | |

| **Easy or**  **very easy** | **1886 (42.6%)** | **3244 (56.4%)** | **3069 (53.3%)** | **2238 (50.5%)** | **1777 (40.1%)** | **2112 (36.7%)** | **2002 (45.2%)** | **2727 (47.4%)** |
| --- | --- | --- | --- | --- | --- | --- | --- | --- |
| **Odds ratio** | 1.83  (95% CI 1.55 to 2.16) | | 1.13  (95% CI 0.96 to 1.33) | | 0.84  (95% CI 0.73 to 0.96) | | 1.08  (95% CI 0.93 to 1.25) | |
| **Adjusted difference** | **15.0%**  (95% CI 10.9% to 19.0%) | | **3.0%**  (95% CI -1.0% to 7.0%) | | **-4.1%**  (95% CI -1.0% to -7.3%) | | **1.9%**  (95% CI -1.8% to 5.6%) | |

* Missing values and don’t know are pooled with difficult and very difficult.

^†^ The difference is an adjusted difference, based on mixed models with a random effects term for the clusters and the stratification variables modelled as fixed effects, using logistic regression. The odds ratios from the logistic regressions have been converted to differences using the intervention schools as the reference and the inverse of the odds ratios shown here.

^‡^ Results based on responses at the end of the term when the intervention was delivered.

**Table S10. Self-reported behaviour - awareness of treatment claims**

*How often do you hear treatment claims?*

|  | **Control schools**  N=2844 | **Intervention schools**  N=3943 |
| --- | --- | --- |
| One or more most days | 572 (20.1%) | 1000 (25.4%) |
| One or more most weeks | 374 (13.2%) | 599 (15.2%) |
| One or more most months | 497 (17.5%) | 715 (18.1%) |
| Almost never | 653 (23.0%) | 788 (20.0%) |
| I don’t know | 717 (25.2%) | 810 (20.5%) |
| Missing | 31 (1.1%) | 31 (0.8%) |
| **One or more most days or most weeks** | **946 (33.8%)** | **1599 (40.6%)** |
| **Odds ratio*** | 1.35  (95% CI 1.02 - 1.79)  P = 0.0356 | |
| **Adjusted difference^†^** | **7.0%**  (95% CI 0.5% to 12.9%) | |

*The odds ratio for the dichotomised data is shown in the table. The odds ratio from the mixed ordinal logistic regression was 1.30 (95% CI 1.01 to 1.67, P = 0.0431).

^†^ The difference is an adjusted difference, based on a mixed model with a random effects term for the clusters and the stratification variables modelled as fixed effects, using logistic regression. The odds ratio from the logistic regression has been converted to a difference using the intervention schools as the reference and the inverse of the odds ratios shown here.

**Table S11. Self-reported behaviour - assessment of trustworthiness of treatment claims**

*How sure are you that the treatment claim you heard is true or can be trusted?*

|  | **Control schools**  N=2844 | **Intervention schools**  N=3943 |
| --- | --- | --- |
| Missing | 49 (1.7%) | 60 (1.5%) |
| Not very sure because I don’t know the reason behind the claim | 665 (23.4%) | 1039 (26.4%) |
| Not very sure because the reason behind the claim was not good | 543 (19.1%) | 1087 (27.6%) |
| Very sure because the reason behind the claim was good | 704 (24.8%) | 790 (20.0%) |
| I don’t know because I don’t know how to decide whether it is true or not | 883 (31.0%) | 967 (24.5%) |
| **Very sure or I don’t know** | **1587 (55.8%)** | **1757 (44.6%)** |
| **Odds ratio (very sure or I don’t know vs other)** | 0.55  (95% CI 0.45 - 0.67)  P<0.0001 | |
| **Adjusted difference*** | **-15.0%**  (95% CI -9.9% to -19.7%) | |
| **Odds ratio (consistent with what they identified as the basis for the claim)^†^** | 1.45  (95% CI 1.18 - 1.75)  P=0.000549 | |
| **Adjusted difference*** | **7.6%**  (95% CI 3.5% - 11.1%) | |

^*^ The differences are adjusted differences, based on mixed models with a random effects term for the clusters and the stratification variables modelled as fixed effects, using logistic regression. The odds ratio from the logistic regression has been converted to a difference using the intervention schools as the reference and the inverse of the odds ratios shown here.

^†^ See Table S11.

**Table S12. Consistent (correct) answers regarding certainty about treatment claims***

| **If you heard about a treatment claim, what was it based on?** | **How sure are you that the treatment claim you heard is true or can be trusted?** |
| --- | --- |
| Someone’s personal experience using the treatment | Not very sure because the reason behind the claim was not good |
| What an expert said about it | Not very sure because the reason behind the claim was not good |
| A research study that compared the treatment with another treatment or no treatment | Not very sure because the reason behind the claim was not good  OR  Very sure because the reason behind the claim was good |
| Something else | Not very sure because the reason behind the claim was not good |
| I could not tell what the treatment claim was based on | Not very sure because I don’t know the reason behind the claim |

* Questions 28.5 and 28.6 in Appendix 1

**Table S13. Self-reported behaviour - assessment of the basis of treatment claims**

*For the last treatment claim that you heard, did you think about what that treatment claim that you heard was based on?*

|  | **Control schools**  N=2844 | **Intervention schools**  N=3943 |
| --- | --- | --- |
| Missing | 50 (1.8%) | 57 (1.4%) |
| No | 512 (18.0%) | 845 (21.4%) |
| Yes | 1387 (48.8%) | 2116 (53.7%) |
| I don’t remember | 895 (31.5%) | 925 (23.5%) |
|  |  |  |
| **Odds ratio (yes versus other)** | 1.18  (95% CI 0.95 - 1.47)  P=0.130 | |
| **Adjusted difference*** | **4.1%**  (95% CI -1.2% - 9.6%) | |

^*^ The difference is an adjusted difference, based on a mixed model with a random effects term for the clusters and the stratification variables modelled as fixed effects, using logistic regression. The odds ratio from the logistic regression has been converted to a difference using the intervention schools as the reference and the inverse of the odds ratios shown here.

**Table S14. Self-reported behaviour - assessment of advantages and disadvantages of treatments**

*How sure are you about the advantages and disadvantages of the [most recent] treatment you used?*

|  | **Control**  **schools**  N=2844 | **Intervention**  **schools**  N=3943 |
| --- | --- | --- |
| A) Not very sure because I don’t know the reasons behind the claims about the good and bad things that treatment makes happen | 531 (18.7%) | 851 (21.6%) |
| B) Not very sure because there was not a good reason behind the claims about the advantages of the treatment | 355 (12.5%) | 549 (13.9%) |
| C) Not very sure because I only know about the advantages of the treatment. I also need to know about the disadvantages | 765 (26.9%) | 992 (25.2%) |
| D) Very sure because there is a good reason behind the claims about the advantages and disadvantages of the treatment | 652 (22.9%) | 929 (23.6%) |
| E) I did not use any treatment | 498 (17.5%) | 590 (15.0%) |
| Missing | 43 (1.5%) | 32 (0.8%) |
| **Odds ratio (C versus any other response)** | 1.05  (95% CI 0.86 - 1.30)  P=0.62 | |
| **Adjusted difference answer C vs else** | **-0.9%**  (95% CI -5.3% - 2.7%) | |
| **Odds ratio (D versus any other response)** | 1.03  (95% CI 0.85 - 1.23)  P=0.79 | |
| **Adjusted difference answer D vs else** | **-0.5%**  (95% CI -3.9% - 2.8%) | |

**Table S15. Subgroup analysis - reading skills**

|  | **Control**  **schools** | **Intervention**  **schools** | **Adjusted difference^†^** | **Odds ratio** | ICC |
| --- | --- | --- | --- | --- | --- |
| **Mean score, %** | | | | | |
|  | N children = 893 | N children = 882 |  |  |  |
| Lacking basic reading skills  (N=1775) | Mean score: 47.2%  (SD 16.4%) | Mean score: 57.1%  (SD 18.1%) | **Mean difference: 11.2%**  (95% CI 8.2% to 14.2%) |  | 0.146 |
|  | N children = 1093 | N children = 1579 |  |  |  |
| Basic reading skills  (N=2672) | Mean score: 55.2%  (SD 16.9%) | Mean score: 67.9%  (SD 16.8%) | **Mean difference: 14.8%**  (95% CI 12.3% to 17.3%) |  | 0.162 |
|  | N children = 858 | N children = 1482 |  |  |  |
| Advanced reading skills  (N=2340) | Mean score: 56.3%  (SD 15.6%) | Mean score: 76.5%  (SD 15.5%) | **Mean difference: 19.4%**  (95% CI 16.9% to 21.9%) |  | 0.117 |
| **Passing score** (> 13 out of 24 correct answers) | | | | | |
|  | N children = 893 | N children = 882 |  |  |  |
| Lacking basic reading skills (N=1775) | 36.6% of children  N=327 | 59.3% of children  N=523 | **28.9% more children**  (95% CI 20.8% to 36.7%) | 0.30  (95% CI 0.20 to 0.43) | 0.144 |
|  | N children = 1093 | N children = 1579 |  |  |  |
| Basic reading skills (N=2672) | 57.0% of children  N=623 | 81.2% of children  N= 1282 | **33.6% more children**  (95% CI 24.0% to 41.9%) | 0.21  (95% CI 0.15 to 0.31) | 0.150 |
|  | N children = 858 | N children = 1482 |  |  |  |
| Advanced reading skills  (N=2340) | 60.0% of children  N=514 | 91.4% of children  N=1355 | **33.4% more children**  (95% CI 25.7% to 42.5%) | 0.13  (95% CI 0.09 to 0.18 ) | 0.098 |
| **Mastery score** (> 20 out of 24 correct answers) | | | | | |
|  | N children = 893 | N children = 882 |  | 0.22 |  |
| Lacking basic reading skills (N=1775) | 3.0 % of children  N=27 | 10,1 % of children  N=89 | **7.7% more children**  (95% CI 5.6% to 8.8%) | (95% CI 0.12 to 0.42) | 0.220 |
|  | N children = 1093 | N children = 1579 |  | 0.15 |  |
| Basic reading skills (n=2672) | 6.5% of children  N=71 | 24.1% of children  N=380 | **19.6% more children**  (95% CI 17.0% to 21.3%) | (95% CI 0.09 to 0.24) | 0.192 |
|  | N children = 858 | N children = 1482 |  | 0.06 |  |
| Advanced reading skills (n=2340) | 4.8% of children  N=41 | 45.1% of children  N=669 | **40.4% more children**  (95% CI 38.2% to 41.9%) | (95% CI 0.04 to 0.09) | 0.139 |

* Because reading skills were measured after the intervention, we have not reported a test of interaction here (see Appendix 3).

^†^ The adjusted difference is based on mixed models with a random effects term for the clusters and the stratification variables modelled as fixed effects, using logistic regression for dichotomous outcomes and linear regression for continuous outcomes. The odds ratios from the logistic regressions for passing scores and mastery scores have been converted to differences using the intervention school proportions and the inverse of the odds ratios shown here.

**Table S16. Differences in reading skills**

| **Reading skills** | **Immediately after the intervention*** | | | **One-year follow-up*** | | | **Change from first to second test*** | | |
| --- | --- | --- | --- | --- | --- | --- | --- | --- | --- |
|  | **Control schools**  N children 4412  n  (%) | **Intervention schools**  N children 5711  n  (%) | **Diff** | **Control schools**  N children 2844  n  (%) | **Intervention schools**  N children 3943  n  (%) | **Diff** | **Control schools** | **Intervention schools** | **Diff** |
| **Lacking basic reading skills** | 2139  (48.5%) | 2224  (38.9%) | -9.5% | 893  (31.4%) | 882  (22.4%) | -9.0% | -17.1% | -16.6% | 0.5% |
| **Basic reading skills** | 1507  (34.2%) | 2155  37.7% | 3.6% | 1093  (38.4%) | 1579  (40.0%) | 1.6% | 4.3% | 2.3% | -2.0% |
| **Advanced reading skills** | 766  (17.4%) | 1332  23.3% | 6.0% | 858  (30.2%) | 1482  (37.6%) | 7.4% | 12.8% | 14.3% | 1.5% |

* Reading skills as measured by first four questions in the test administered at the end of the term when the intervention was delivered and the same test one year later. The differences (Diff) are shown between the intervention and control schools for each time the test was administered and the change from the first to the second time.

**Table S17. Subgroup analysis - parent who listened to the podcast**

|  | **Control**  **schools** | **Intervention**  **schools** | **Adjusted effect of the interaction*** |
| --- | --- | --- | --- |
|  | N children = 69 | N children = 98 | **Mean difference: 3.8%**  (95% CI -3.9% to 11.4%)  P=0.3443 |
| Parent in control group  (N=167) | Mean score: 55.1%  (SD 16.4%) | Mean score: 64.5%  (SD 20.2%) |  |
|  | N children = 64 | N children = 104 |  |
| Parent in podcast group  (N=168) | Mean score: 53.6%  (SD 15.9%) | Mean score: 66.3%  (SD 18.6%) |  |

*Adjusted for location, ownership and random effect of clustering, ICC=0.185

**Table S18. Exploratory analyses - p-values for differences between first (end of intervention term) and second (one-year follow-up) effects**

|  | **Control schools** | **Intervention schools** | **Adjusted difference*** | Odds ratio* |
| --- | --- | --- | --- | --- |
| **Primary outcome** |  |  |  |  |
| **Mean score (%)** |  |  |  |  |
| **1. End of intervention term** | Mean score: 43.1%  (SD 15.2%) | Mean score: 62.4%  (SD 18.8%) | 20.0%  (95% CI 17.3% to 22.7%) |  |
| **2. One-year follow-up** | Mean score: 53.0%  (SD 16.8%) | Mean score: 68.7%  (SD 18.2%) | 16.7%  (95% CI 13.9% to 19.5%) |  |
| **Difference between first and second tests** |  |  | **-6.9%**  **(95% CI 6.5 to 7.3)**  **P<0.00001** |  |
| **Passing score****^†^** |  |  |  |  |
| **1. End of intervention term** | 26.8 % of children  (N=1186/4430) | 69.0 % of children  (N=3967/5753) | 49.8% more children  (95% CI 43.8% to 54.6%) | 9.34  (95% CI 6.62 to 13.18) |
| **2. One-year follow-up** | 51.5 % of children  (N=1464/2844) | 80.1 % of children  (N=3160/3943) | 39.5% more children  (95% CI 29.9% to 47.5%) | 5.88  (95% CI 4.00 to 8.33) |
| **Difference between first and second tests** |  |  |  | **0.36**  **(95% CI: 0.33 to 0.39)**  **P<0.00001** |
| **Secondary outcomes** |  |  |  |  |
| **Mastery score****^‡^** |  |  |  |  |
| **1. End of intervention term** | 0.9% of children  (N=38/4430) | 18.6% of children  (N=1070/5753) | 18.0% more children  (95% CI 17.5% to 18.2%) | 35.33  (95% CI 20.58 to 60.67) |
| **2. One-year follow-up** | 4.9% of children  (N=139/2844) | 28.9% of children  (N=1138/3943) | 25.0% more children  (95% CI 23.2% to 26.5%) | 10.00  (95% CI 6.67 to 16.67) |
| **Difference between first and second tests** |  |  |  | **0.42**  **(95% CI: 0.37 to 0.47)**  **P<0.00001** |

* The differences between effects (adjusted mean differences and odds ratios) from is based on mixed models with a random effects term for the clusters (schools), individuals (who are used twice in these analyses), and the stratification variables modelled as fixed effects, using logistic regression for dichotomous outcomes and linear regression for continuous outcomes.

^†^ 13 or more out of 24 correct answers

^‡^ 20 or more out of 24 correct answers

**Table S19. Exploratory analyses excluding children who did not take the test both times**

|  | **Control schools** | **Intervention schools** | **Adjusted difference*** | Odds ratio* | ICC |
| --- | --- | --- | --- | --- | --- |
| **Primary outcome** |  |  |  |  |  |
| **One-year follow-up**  Mean score, % | Mean score: 53.0%  (SD 16.8%) | Mean score: 68.7%  (SD 18.2%) | **Mean difference: 16.7%**  (95% CI 13.9% to 19.5%)  P <0.00001 |  | 0.18 |
| **End of intervention term excluding children who did not take the one-year follow-up test**  Mean score, % | Mean score: 43.8%  (SD 15.5%)  (N=2733) | Mean score: 64.6%  (SD 18.5%)  (N=3875) | Mean difference: 21.6% (95% CI 18.9 – 24.4)  P<0.00001 |  | 0.17 |
| End of intervention term  Mean score, % | Mean score: 43.1%  (SD 15.2%) | Mean score: 62.4%  (SD 18.8%) | Mean difference: 20.0%  (95% CI 17.3% to 22.7%) |  | 0.18 |
| **One-year follow-up**  Passing score  (> 13 out of 24 correct answers) | 51.5 % of children  (N=1464/2844) | 80.1 % of children  (N=3160/3943) | **39.5% more children**  (95% CI 29.9% to 47.5%) | 5.88  (95% CI 4.00 to 8.33)  P <0.00001 | 0.20 |
| **End of intervention term excluding children who did not take the one-year follow-up test**  Passing score  (> 13 out of 24 correct answers) | 28.4%  (776/2733) | 74.0%  2867/(3875) | **54.1% (95% CI: 47.8 – 59.1)**  **P<0.00001** | 0.09 (0.06 – 0.12) | 0.19 |
| End of intervention term  Passing score  (> 13 out of 24 correct answers) | 26.8 % of children  (N=1186/4430) | 69.0 % of children  (N=3967/5753) | 49.8% more children  (95% CI 43.8% to 54.6%) | 9.34  (95% CI 6.62 to 13.18) | 0.19 |
| **Secondary outcomes** |  |  |  |  |  |
| **One-year follow-up**  Mastery score  (> 20 out of 24 correct answers) | 4.9% of children  (N=139/2844) | 28.9% of children  (N=1138/3943) | **Mean difference: 25.0%**  **(23.2%-26.5%)** | 10.00  (95% CI 6.67 to 16.67)  P <0.00001 | 0.19 |
| **End of intervention term excluding children who did not take the one-year follow-up test**  Mastery score  (> 20 out of 24 correct answers) | 0.8%  (N 23/2733) | 21.9%  (N 847/3875) | **21.2% (95% CI 20.7 – 21.5)** | 0.02 (0.01 – 0.04)  P<0.00001 | 0.16 |
| End of intervention term  Mastery score  (> 20 out of 24 correct answers) | 0.9% of children  (N=38/4430) | 18.6% of children  (N=1070/5753) | **18.0% more children**  (95% CI 17.5% to 18.2%) | 35.33  (95% CI 20.58 to 60.67) | 0.21 |
