# Supplementary material for: Effects of the Informed Health Choices primary school intervention on the ability of children in Uganda to assess the reliability of claims about treatment effects, 1-year follow-up: a cluster-randomised trial
Source: Trials. 2020 Jan 6;21:27. doi: 10.1186/s13063-019-3960-9 (PMC6945419; doi:10.1186/s13063-019-3960-9)

Follow-up one year after the intervention was delivered

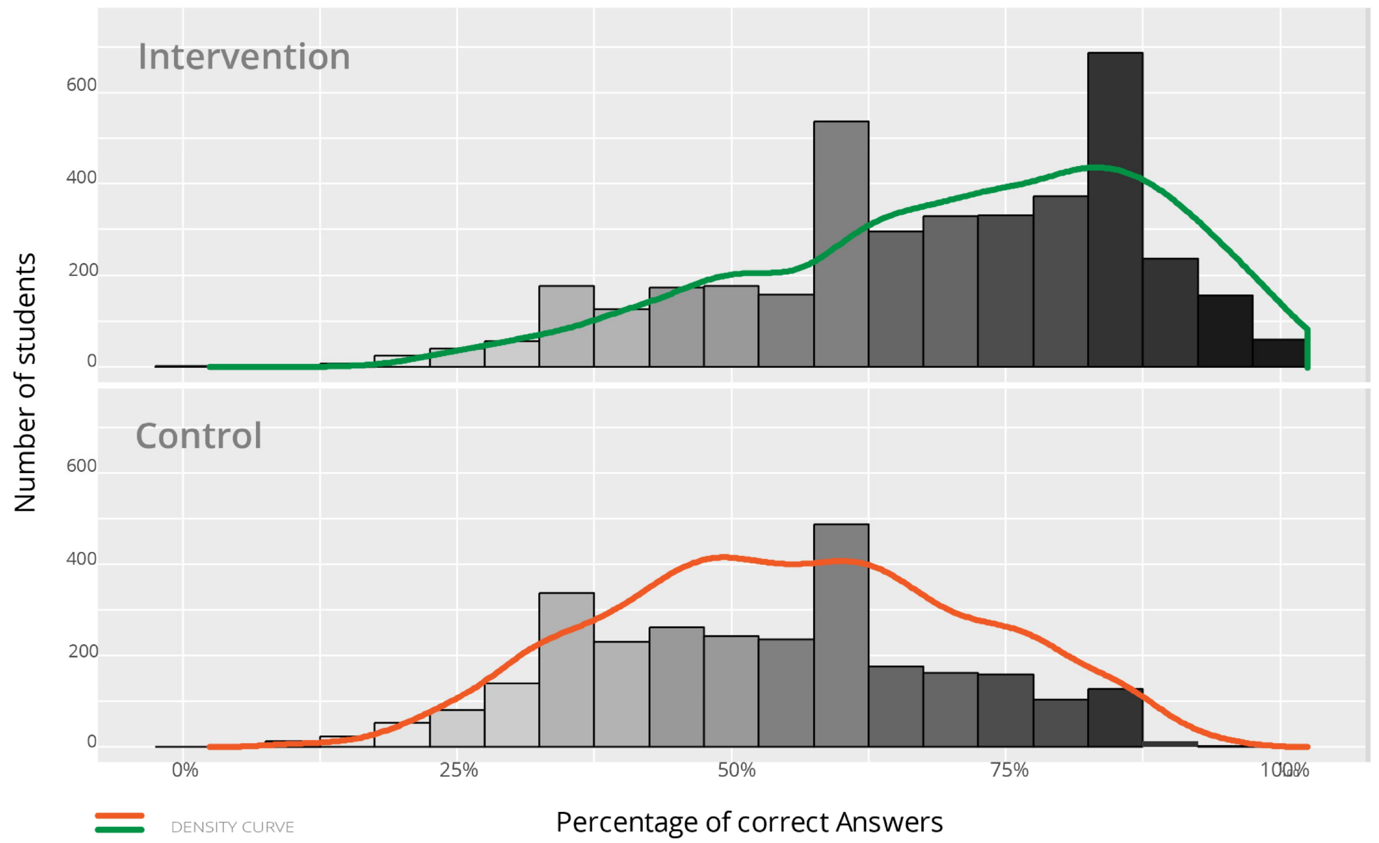

End of term when the intervention was delivered

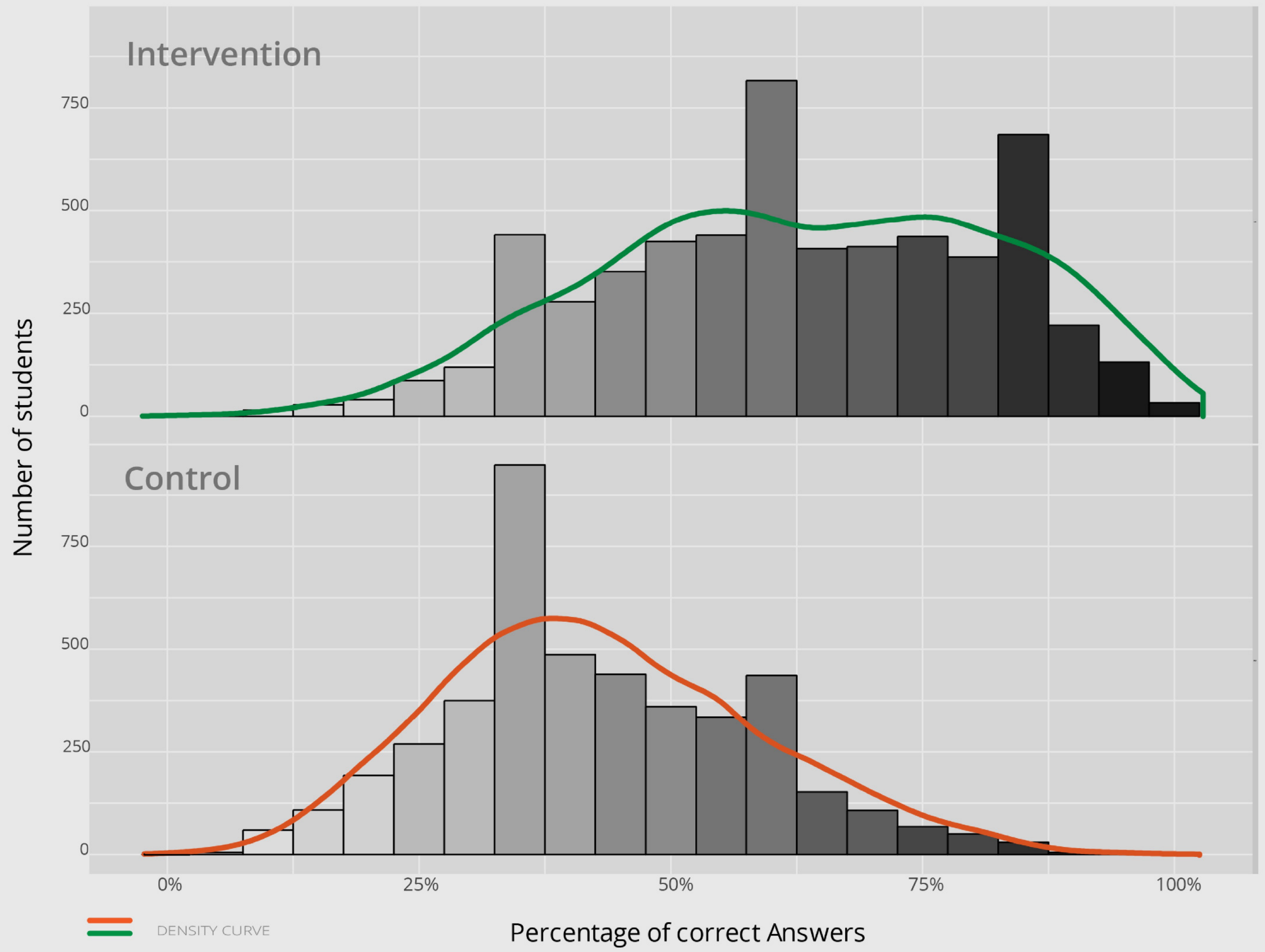

Supplement: Supplementary file 3 — Additional file 3. Distribution of scores and curves. [file 13063_2019_3960_MOESM3_ESM.pdf]
